# Antibody dependent cellular cytotoxicity-inducing anti-EGFR antibodies as effective therapeutic option for cutaneous melanoma resistant to BRAF inhibitors

**DOI:** 10.3389/fimmu.2024.1336566

**Published:** 2024-03-06

**Authors:** Elena Muraro, Barbara Montico, Benedict Lum, Francesca Colizzi, Giorgio Giurato, Annamaria Salvati, Roberto Guerrieri, Aurora Rizzo, Elisa Comaro, Vincenzo Canzonieri, Andrea Anichini, Michele Del Vecchio, Roberta Mortarini, Massimo Milione, Alessandro Weisz, Maria Antonietta Pizzichetta, Fiona Simpson, Riccardo Dolcetti, Elisabetta Fratta, Luca Sigalotti

**Affiliations:** ^1^ Immunopathology and Cancer Biomarkers, Department of Translational Research, Centro di Riferimento Oncologico di Aviano (CRO) IRCCS, Aviano, Italy; ^2^ Frazer Institute, The University of Queensland, Brisbane, QLD, Australia; ^3^ Laboratory of Molecular Medicine and Genomics, Department of Medicine, Surgery and Dentistry “Scuola Medica Salernitana”, University of Salerno, Baronissi, Italy; ^4^ Genome Research Center for Health - CRGS, Baronissi, Italy; ^5^ Molecular Pathology and Medical Genomics Program, AOU ‘S. Giovanni di Dio e Ruggi d’Aragona’ University of Salerno and Rete Oncologica Campana, Salerno, Italy; ^6^ Division of Pathology, Centro di Riferimento Oncologico di Aviano (CRO) IRCCS, Aviano, Italy; ^7^ Department of Medical, Surgical and Health Sciences, University of Trieste, Trieste, Italy; ^8^ Human Tumors Immunobiology Unit, Department of Research, Fondazione IRCCS Istituto Nazionale dei Tumori, Milan, Italy; ^9^ Melanoma Unit, Department of Medical Oncology, Fondazione IRCCS Istituto Nazionale dei Tumori, Milan, Italy; ^10^ Pathology Unit 1, Department of Pathology and Laboratory Medicine, Fondazione IRCCS Istituto Nazionale dei Tumori, Milan, Italy; ^11^ Division of Medical Oncology, Centro di Riferimento Oncologico di Aviano (CRO) IRCCS, Aviano, Italy; ^12^ Department of Dermatology, University of Trieste, Trieste, Italy; ^13^ Translational and Clinical Immunotherapy, Peter MacCallum Cancer Centre, Melbourne, VIC, Australia; ^14^ Sir Peter MacCallum Department of Oncology, The University of Melbourne, Melbourne, VIC, Australia; ^15^ Department of Microbiology and Immunology, The University of Melbourne, Melbourne, VIC, Australia; ^16^ Faculty of Medicine, The University of Queensland, Brisbane, QLD, Australia; ^17^ Oncogenetics and Functional Oncogenomics Unit, Centro di Riferimento Oncologico di Aviano (CRO) IRCCS, Aviano, Italy

**Keywords:** cutaneous melanoma, BRAF, drug resistance, receptor tyrosine kinases, antibody dependent cell cytotoxicity

## Abstract

**Introduction:**

About 50% of cutaneous melanoma (CM) patients present activating BRAF mutations that can be effectively targeted by BRAF inhibitors (BRAFi). However, 20% of CM patients exhibit intrinsic drug resistance to BRAFi, while most of the others develop adaptive resistance over time. The mechanisms involved in BRAFi resistance are disparate and globally seem to rewire the cellular signaling profile by up-regulating different receptor tyrosine kinases (RTKs), such as the epidermal growth factor receptor (EGFR). RTKs inhibitors have not clearly demonstrated anti-tumor activity in BRAFi resistant models. To overcome this issue, we wondered whether the shared up-regulated RTK phenotype associated with BRAFi resistance could be exploited by using immune weapons as the antibody-dependent cell cytotoxicity (ADCC)-mediated effect of anti-RTKs antibodies, and kill tumor cells independently from the mechanistic roots.

**Methods and results:**

By using an *in vitro* model of BRAFi resistance, we detected increased membrane expression of EGFR, both at mRNA and protein level in 4 out of 9 BRAFi-resistant (VR) CM cultures as compared to their parental sensitive cells. Increased EGFR phosphorylation and AKT activation were observed in the VR CM cultures. EGFR signaling appeared dispensable for maintaining resistance, since small molecule-, antibody- and CRISPR-targeting of EGFR did not restore sensitivity of VR cells to BRAFi. Importantly, immune-targeting of EGFR by the anti-EGFR antibody cetuximab efficiently and specifically killed EGFR-expressing VR CM cells, both *in vitro* and in humanized mouse models *in vivo*, triggering ADCC by healthy donors’ and patients’ peripheral blood cells.

**Conclusion:**

Our data demonstrate the efficacy of immune targeting of RTKs expressed by CM relapsing on BRAFi, providing the proof-of-concept supporting the assessment of anti-RTK antibodies in combination therapies in this setting. This strategy might be expected to concomitantly trigger the crosstalk of adaptive immune response leading to a complementing T cell immune rejection of tumors.

## 1 Introduction

Cutaneous melanoma (CM) is a very aggressive malignancy that originates from melanocytes, and shows a continuously increasing incidence in industrialized countries, with a more frequent diagnosis in young and middle-aged adults (1). Early tumor recognition and subsequent surgical treatment are usually curative for CM. However, diagnosis of CM may be difficult and it may be clinically misdiagnosed in a significant number of cases (2). CM escaping the clinical recognition frequently present in an advanced stage, are essentially unresponsive to conventional therapies, and show a poor prognosis (3, 4). Efforts in defining the biology of this malignancy have identified activating BRAF mutations in about 50% of CM patients. The constitutive activation of MAPK signaling caused by mutant BRAF appears a major driver of CM proliferation, survival, and progression. Accordingly, small molecule inhibitors of BRAF (BRAFi) demonstrated important clinical activities in BRAF-mutant CM, with remarkable response rates, and a significantly improved progression-free and overall survival in the advanced disease (5–9). However, the clinical effectiveness of these targeted therapeutics is greatly impaired by the almost invariable onset of an early drug resistance, which leads to tumor progression within about 7 months from the start of treatment in 50% of patients (8, 10, 11). Besides, about 20% of CM patients show intrinsic resistance to BRAFi and do not respond to treatment (12). The underlying mechanisms of resistance so far described are various and heterogeneous (7, 13–23).

Though the underlying mechanisms are disparate, a unifying feature of CM resistance to BRAFi appears the rewiring cellular signaling profiles (18, 20, 21, 24–28), which is frequently associated with a *de novo*, up-regulated or “positively selected” expression of different receptor tyrosine kinases (RTKs), including AXL, EGFR, IGF-1R, PDGFRα, and PDGFRβ (18–20, 24, 29–32). In this context, whether the expression of specific RTK is essential for the maintenance of the resistant phenotype is not fully defined. Indeed, literature data are discrepant, including reports showing cooperation between small molecule RTK inhibitors (RTKi) (e.g., the EGFR inhibitor gefitinib, the AXL inhibitor R428) and BRAFi [e.g. PLX4032 (vemurafenib), PLX4720] in reducing the *in vitro* and *in vivo* growth of BRAFi-resistant CM cells (19, 29, 30, 33–35), together with studies reporting negligible activity of RTKi on sensitivity to BRAFi after resistance is acquired (20, 24, 36). In addition, a recent study by Molnar et al. indicated that BRAFi-resistant CM cells with higher EGFR expression were more resistant to the treatment with erlotinib respect to those that expressed low levels (37). Besides, EGFR inhibitors simultaneously administered with other agents (38) or inhibitors of common RTK downstream pathways, such as those targeting SRC (e.g. Dasatinib) or PI3K (e.g. GDC0941), were shown to be more effective in re-sensitizing to BRAFi (7, 20, 29, 36, 39), suggesting that the signaling alterations responsible for the resistant phenotype are broad, and thus, likely not strictly dependent on the activity of a single RTK. In line with these notions, recent single cell CM sequencing approaches are suggesting the co-existence in the same tumor of very different populations that are resistant to targeted therapies still being characterized by importantly dissimilar cellular programs, while pre-existing rare populations of CM cells marked by surface EGFR expression have been proposed as possible seeds for relapsing tumors (18).

These complex redundant signaling networks that are emerging as the main drivers of BRAFi resistance appear difficult to target by mechanistic approaches, and likely amenable to further resistance by signaling rewiring. Nevertheless, the shared phenotype of BRAFi resistant cells may itself represent a therapeutic target that could be actionable by drugs already available in the clinic. Among these, monoclonal antibodies (mAbs) directed to RTK appear particularly suited and appealing as compared to small molecule RTKi, since mAbs are able to act also via immune-mediated mechanisms. Indeed, when antibodies bind antigens exposed on cells, their Fc region can ligate and crosslink the Fcγ receptor (FcγR) expressed on immune effector cells, mainly Natural Killer (NK) cells, but also myeloid-derived effectors (40). Upon FcγR engagement, effector cells are activated and release cytokines as well as cytotoxic granules that ultimately lead to target cell killing in a process referred to as Antibody-Dependent Cell-mediated Cytotoxicity (ADCC) (40). In this process, antibodies of the IgG1 isotype (e.g., the anti-CD20 rituximab, the anti-HER2 trastuzumab, the anti-EGFR cetuximab) are particularly effective (41).

From a therapeutic point of view, the immune-mediated activities of anti-RTK mAbs were shown to significantly contribute to their clinical activity. This is well established for trastuzumab, both in pre-clinical and clinical settings (42–44), and recent data support a role of ADCC also in delivering part of the clinical activity of cetuximab (45). Indeed, several immunogenetic studies associated high-affinity FcγR genotypes to improved therapeutic efficacy of cetuximab in colorectal cancer patients (46–49), and the ability of patients’ NK cells to mount *ex vivo* an effective cetuximab-triggered ADCC associated with an improved clinical benefit of colorectal and head and neck squamous cell carcinoma patients (49, 50). Notably, ADCC activity could allow therapeutic targeting of RTK-expressing cells independently of the effects on signaling, contributing to trigger a broader anti-tumor immune response as well (44, 51, 52).

Based on the above notions, we sought to comprehensively investigate the feasibility of targeting RTKs, in particular EGFR, as a strategy to treat CM patients relapsing on BRAFi. A panel of 9 BRAF mutant metastatic CM cell cultures, which were made resistant to the PLX4032 BRAFi, was used as a model to evaluate: i) the expression of RTK and their ligands in CM cells acquiring BRAFi resistance; ii) the effect of EGFR targeting by small molecule inhibitors, mAbs, and genomic editing approaches on BRAFi sensitivity; and iii) the anti-tumor efficacy of EGFR targeting mAbs through ADCC.

Our data demonstrate that, in our model, signaling through EGFR-up-regulation/*de novo* expression is not required for the maintenance of BRAFi resistance, and, as such, inhibition of signaling *via* single RTK may fail in restoring sensitivity to BRAFi. Nevertheless, EGFR targeting through immune-mediated mechanisms is effective in killing tumor cells by ADCC, both *in vitro* and *in vivo*. These data support the potential clinical activity of anti-RTK mAbs in BRAFi-resistant CM and provide the grounds for their clinical evaluation in combination therapies for the treatment of CM patients relapsing on BRAFi.

## 2 Results

### 2.1 Expression of RTKs and their ligands in CM cell cultures with acquired resistance to BRAFi

**Figure 1:**
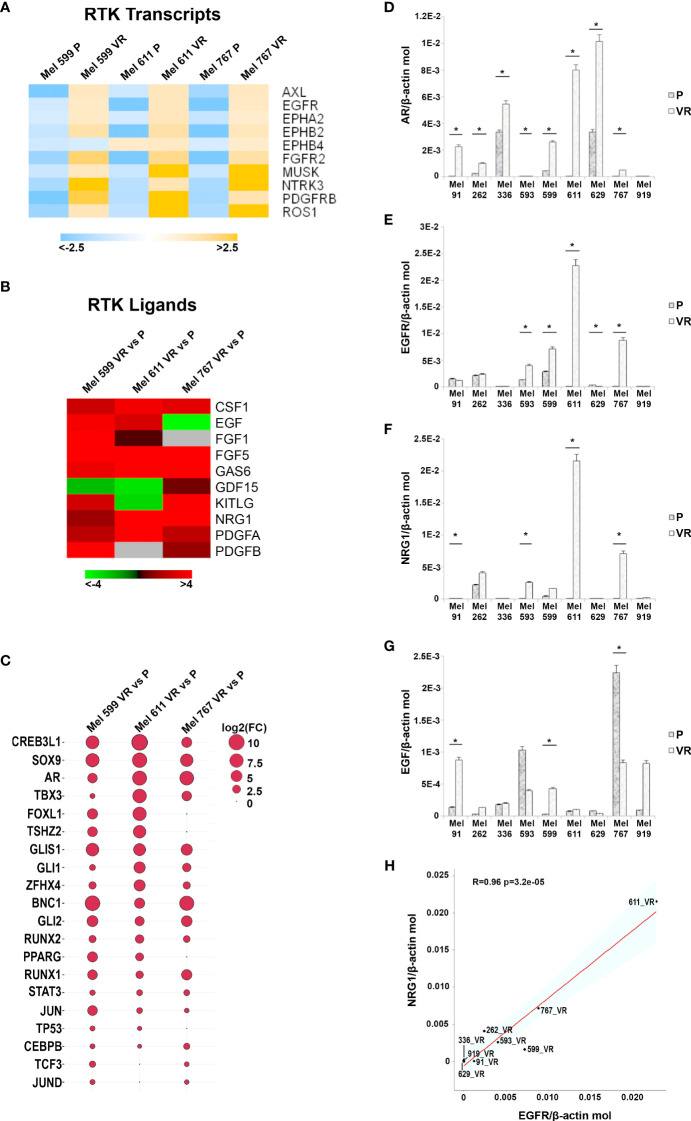
Transcripts analysis in P and VR CM cells. **(A)** Heatmap summarizing expression data for the differentially expression RTK transcripts. Data are shown as normalized expression values in log2 scale and centered on the median value. **(B)** Heatmap showing the Fold-Change values of RTK ligands. **(C)** Ballon-plot representing the Fold-Change values of the putative transcription factors regulating the RTK transcripts. The dimension of the ballon is correlated to the Fold-Change value. **(D–G)** Total RNA was extracted from P and VR cell cultures, retro-transcribed and subjected to SYBR Green quantitative real-time PCR analysis using assays for transcripts encoding AR, EGFR and its ligands NRG1 and EGF, as well as the housekeeping gene β-actin. Level of gene expression is reported as number of molecules of the target gene normalized to the number of β-actin molecules. Data are presented as mean + standard deviation of values obtained from at least 3 independent experiments; * p ≤ 0.05. **(H)** Plot showing the Pearson correlation between NRG1 and EGFR mRNA values. The Student’s t-test was used to compute the p-value.

**Figure 2:**
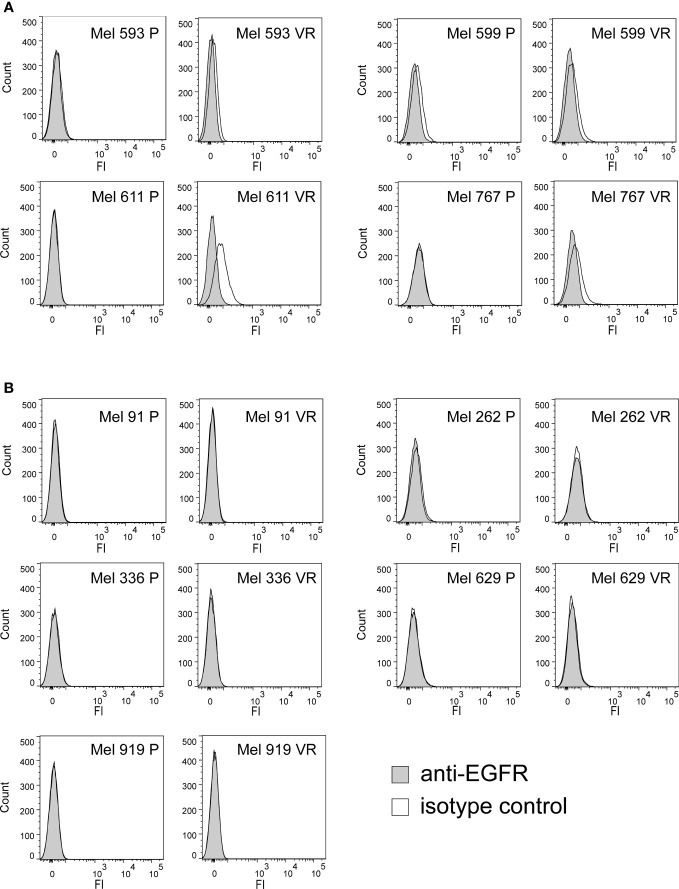
Cell surface expression of EGFR on P and VR CM cells. The cell-surface expression of EGFR was evaluated on P and VR isogenic cell cultures by flow cytometry. Filled gray histograms refer to isotype labeled cells; black empty histograms refer to cells labeled with anti-EGFR antibody. **(A)** Pairs of isogenic cultures where VR cells show surface EGFR expression, either acquired following gaining of resistance or constitutively present also on P cells. **(B)** Pairs of isogenic cultures where VR cells have no surface EGFR expression. Y axis, counts; X axis, fluorescence intensity.

**Figure 3:**
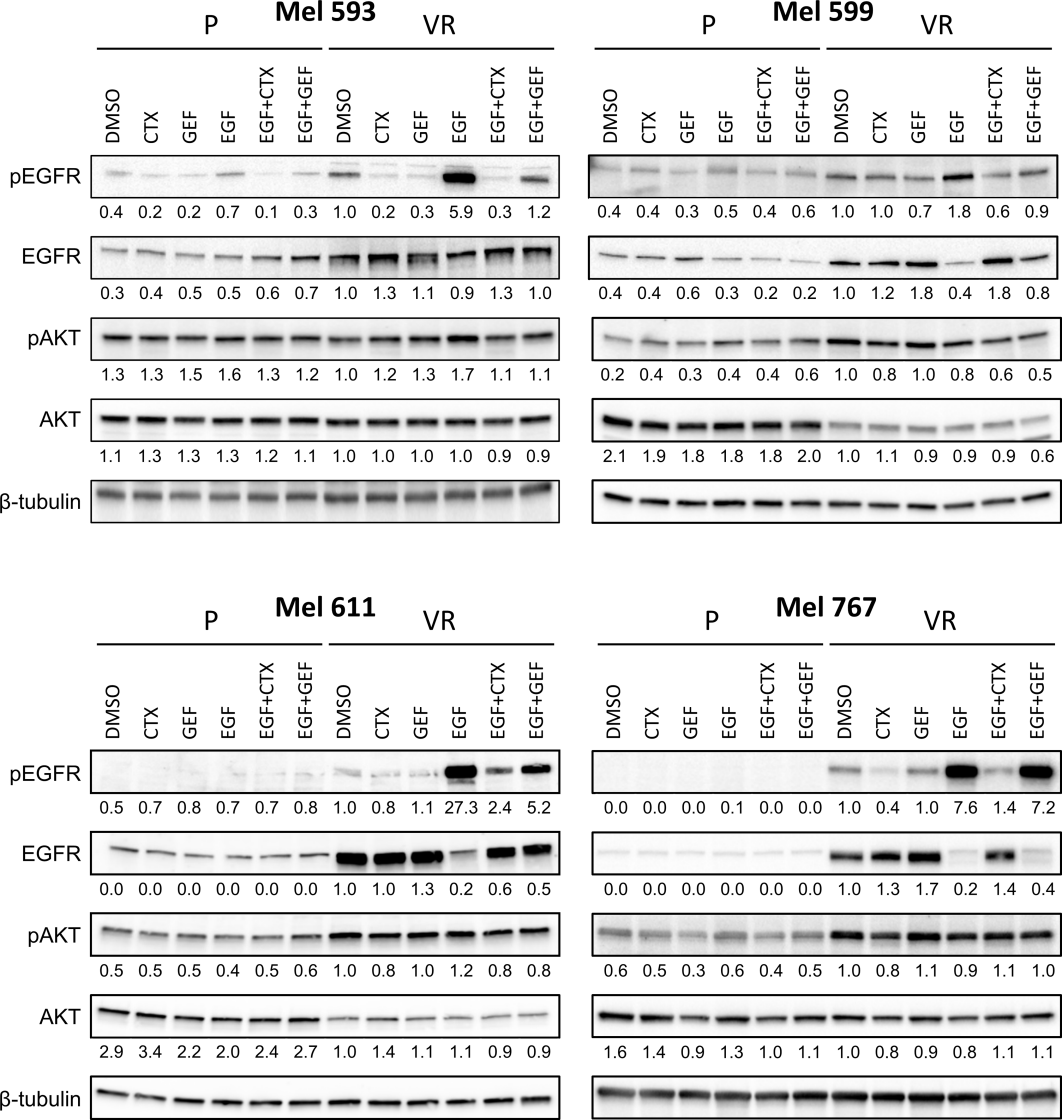
EGFR signaling in P and VR cell cultures. P and VR Mel 593, Mel 599, Mel 611, and Mel 767 CM cells were seeded in 6 well plates and treated for 24 h with either DMSO, 20 μg/ml cetuximab (CTX) or 2.5 μM gefitinib (GEF), with or without the addition of 20 ng/ml EGF. VR cells were maintained without PLX4032 for the duration of the assay. Immunoblotting was performed on cell lysates to evaluate EGFR signaling by EGFR and AKT phosphorylation (pEGFR, pAKT). Total EGFR and AKT (EGFR, AKT) served as reference, β-tubulin served as loading control. Quantifications are reported under the panels, and represent the densitometry values of the protein normalized to those of β-tubulin and referred to VR DMSO set to 1. Representative blots of at least 3 independent experiments.

In an attempt to generate an *in vitro* model of resistance to BRAFi, resistant cell cultures (vemurafenib resistant, VR) were obtained from 9 BRAF-mutant metastatic CM cell lines through sequential adaptation to escalating concentrations of the BRAFi PLX4032. Parental cell lines (P) carried BRAF V600E or BRAF V600K activating BRAF mutations and were all wild-type for NRAS (**Supplementary Table 1**). Dose-response curves confirmed an extreme resistance of VR cells to PLX 4032 (IC_50_ ranging from 16.7 µM of Mel 262 to 29.6 µM of Mel 599) as compared to their highly sensitive P counterparts (IC_50_ ranging from 0.06 µM of Mel 593 to 0.44 µM of Mel 336) (**Supplementary Figure 1**). RNA sequencing was then performed on three cell lines (Mel 599, Mel 611 and Mel 767) in order to define changes in RTKs expression profile in VR cells (**Supplementary Table 2**). As shown in **Figure 1**, different RTKs and several ligands were *de novo* expressed/up-regulated in VR cell lines (**Figures 1A, B**). In addition, an augmented expression of a number of transcription factors that have been described as positive determinants of BRAFi resistance was also observed (**Figure 1C**). Among them, a consistent increase in androgen receptor (AR) expression was found in VR cells. Interestingly, AR has been recently described as a positive determinant of BRAFi resistance and EGFR expression (53). Hence, all VR cells were quantitatively evaluated for AR and EGFR transcripts. An up-regulation of AR expression was found in all but one VR cell lines (**Figure 1D**, **Supplementary Table 3**), whereas a significant increase in EGFR mRNA expression levels was detected in 4 (Mel 593, Mel 599, Mel 611 and Mel 767) out of the 9 VR cell lines investigated, as compared to their P isogenic counterpart (**Figure 1E**, **Supplementary Table 3**), thus suggesting that AR might be partially responsible for EGFR overexpression in our *in vitro* model. EGFR transcript up-regulation was paralleled by that of its NRG1 ligand (**Figures 1F, H**, **Supplementary Table 3**), while no consistent co-expression was found with the other ligands tested (i.e. EGF) (**Figures 1G**, **Supplementary Table 3**). In line with molecular data, flow cytometry analyses confirmed a *de novo* cell-surface expression of EGFR in VR Mel 593, Mel 611 and Mel 767 cells as compared to their respective P cells, while a constitutive EGFR surface expression was found in both P and VR Mel 599 cells (**Figure 2**). The increased EGFR expression in VR cells lines observed by flow cytometry was then confirmed by the results of western blotting analyses (**Figure 3**). The above information is of particular value, suggesting that EGFR expressed on the cell membrane following establishment of BRAFi resistance could be targeted by EGFR-specific antibodies. Notably, the analysis of TCGA data confirmed a significantly higher expression of transcripts of several RTKs and RTK ligands, including EGFR and NRG1, as well as transcription factors positively associated to BRAFi resistance in CM samples predicted to be intrinsically resistant to BRAFi (**Supplementary Figure 2**) based on a previously described scoring (33).

### 2.2 Signaling correlates of *de novo* EGFR expression in CM cells with acquired resistance to PLX 4032

Although the molecular mechanisms responsible for the resistance have not been investigated in this study, to evaluate whether the *de novo* expression of EGFR in VR cells was associated with modifications in cell signaling that could contribute to the maintenance of the PLX4032-resistant phenotype, Western blot analyses were carried out on P and matched VR cells (**Figure 3**, **Supplementary Figure 3**). Constitutive EGFR phosphorylation was heterogeneous, being evident in Mel 767 VR, Mel 599 VR and Mel 593 VR, while very limited in Mel 611 VR cells. EGFR phosphorylation following addition of exogenous EGF was triggered in all VR cells examined. Notably, exogenous EGF slightly upregulated EGFR phosphorylation also in Mel 599 P cells, suggesting that the EGFR constitutively expressed by these cells is functional. EGF stimulation triggered downregulation of EGFR, in line with literature data reporting degradation of activated EGFR (54–61). Independent of the degree of constitutive EGFR phosphorylation, activation of the AKT pathway emerged as a common feature of VR cells, as demonstrated by the increased phosphorylation of AKT1 found in 3 out of 4 VR cell cultures as compared to their P isogenic cells. Intriguingly, exogenous EGF did not further increase AKT1 phosphorylation (**Figure 3**), suggesting that the constitutively hyper activated AKT pathway in VR cells is not amenable to additional significant activation by EGFR stimulation (**Figure 3**, **Supplementary Figure 3**), similarly to what reported in the literature in other settings (62–64). Accordingly, significant enrichment in PI3K/AKT and EGFR signaling pathways was observed both in VR cell lines and in CM samples predicted to be intrinsically resistant to BRAFi (**Supplementary Figure 2**).

### 2.3 Activity of small molecule or antibody EGFR inhibitors on sensitivity to BRAFi

**Figure 4:**
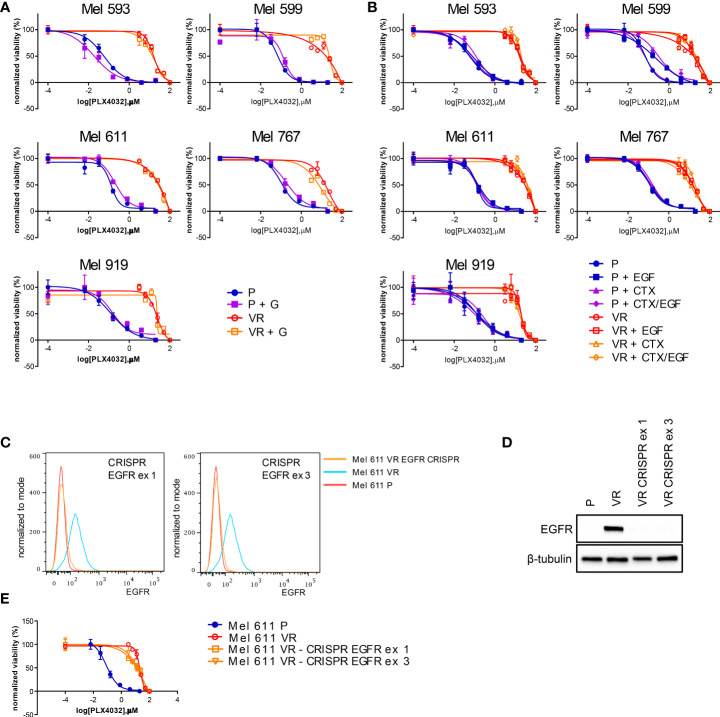
PLX4032 sensitivity of CM cells following EGFR targeting by small molecules, antibodies or genetic editing. P or VR CM cells were seeded in 96 well plates and treated for 72h with scalar doses of PLX4032, with or without the addition of 2.5 μM of the EGFR small molecule inhibitor gefitinib (GEF) **(A)**, or 20 μg/ml of the anti-EGFR mAb cetuximab (CTX), in the presence or absence of 20 ng/ml EGF **(B)**. **(C, D)** the expression of EGFR protein in Mel 611 VR cells was knocked down by CRISPR-CAS9 targeting EGFR at either exon 1 or exon 3. EGFR-negative Mel 611 VR cells were viably sorted by FACS and used for further assays. **(C)** Flow cytometry analysis confirming absence of cell surface EGFR expression on Mel 611 VR CRISPR-edited (orange line) at EGFR exon 1 (left panel) or exon 3 (right panel). **(D)** Western blot analysis confirming absence of EGFR protein in Mel 611 VR CRISPR-edited. **(E)** Dose-response curves to PLX4032 of Mel 611 P, VR and VR CRISPR-edited at EGFR exon 1 (Mel 611 VR - CRISPR EGFR ex1) or exon 3 (Mel 611 VR - CRISPR EGFR ex3). Cells were seeded in 96 well plates and treated for 72h with scalar doses of PLX4032. For all dose-response experiments, cell viability was evaluated using standard 4h MTT assays. Dose-response curves report means and standard deviations of three replicates vs. logarithmic scale of PLX4032 concentration. Normalized viability (%) is relative to vehicle-treated cells.

On the grounds of the above information, and of literature data involving EGFR signaling in resistance to BRAFi, we evaluated whether VR cells became “addicted” to EGFR oncogenic activity. To this end, VR cells were treated with either the small molecule EGFR inhibitor gefitinib or the anti-EGFR antibody cetuximab, which are able to inhibit EGFR phosphorylation through different mechanisms (65, 66). The extent of EGFR signaling inhibition induced by these drugs correlated with the level of constitutive EGFR activation, with cetuximab being effective in reducing the constitutive EGFR phosphorylation in Mel 767 VR cells, while having limited effect on Mel 593, Mel 599 and Mel 611 VR cultures. On the other hand, both gefitinib and cetuximab were able to counteract EGFR phosphorylation triggered by exogenous EGF, as clearly observed in Mel 599 and Mel 611 VR, and to a lesser extent in Mel 593, with cetuximab delivering the strongest activity (**Figure 3**, **Supplementary Figure 3**). Intriguingly, neither gefitinib nor cetuximab reduced AKT1 phosphorylation, suggesting a role for different or additional pathways in sustaining this activation rather than being completely or mainly relying on EGFR activation. In line with this observation, and with the hypothesis that AKT pathway activation could contribute to the acquired resistance to BRAFi, we observed that the addition of either gefitinib or cetuximab was unable to restore the sensitivity of VR cells to PLX-4032, as evaluated by dose-response curves measuring cell viability of P and VR cells cultured in the presence of escalating concentrations of PLX-4032 (**Figures 4A, B**). Similar results were found when sensitivity to PLX-4032 was evaluated in clonogenic assays in the presence or absence of gefitinib or cetuximab (**Supplementary Figure 4**). To evaluate whether EGFR might have a role in sustaining PLX-4032 resistance in our model, EGFR expression in Mel 611 VR was knocked down by using the CRISPR/CAS9 technology (**Figures 4C, D**), and the resulting cells were evaluated for sensitivity to PLX-4032. As shown in **Figure 4E**, EGFR-negative Mel 611 VR cells retained a complete resistance to PLX-4032, showing a dose-response curve superimposable to that of the un-edited Mel 611 VR cells, thus confirming that EGFR expression can be dispensable for maintaining the BRAFi resistant phenotype.

### 2.4 Targeting EGFR-positive BRAFi-resistant CM cells by ADCC-mediating anti-EGFR antibodies

**Figure 5:**
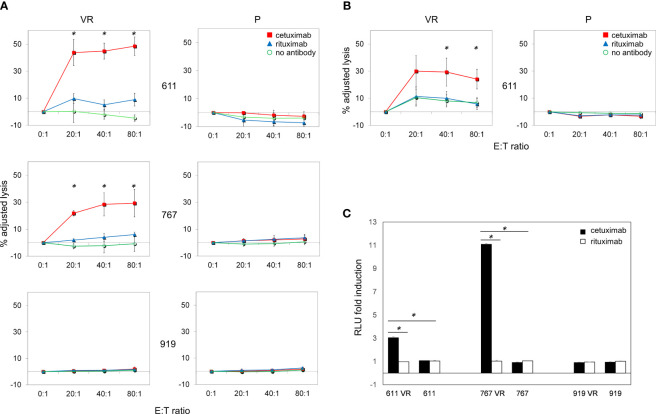
Cetuximab-mediated ADCC on P and VR CM cells. Antibody-Dependent Cell Cytotoxicity (ADCC) mediated by cetuximab in CM cell lines. **(A)** Representative graphs of ADCC mediated by cetuximab (red square) in P and VR CM cell lines. Data are shown as mean and standard deviation of 3 independent experiments performed with PBMCs obtained from 3 different healthy donors at 4 different effector:target ratios (80:1, 40:1, 20:1, 0:1), co-cultured overnight with target cells at 37°C and 5% of CO_2_. ADCC efficiency is expressed as adjusted lysis, calculated as 100-adjusted survival, i.e. 100*(survival with effectors/survival without effectors). The specific lysis values measured in the presence of rituximab (blue triangle) and in the absence of antibodies (green circle) are considered as negative controls. The X axis reported the different effector:target (E:T) ratios used. **(B)** ADCC efficiency mediated by PBMCs obtained from 3 different CM patients. **(C)** ADCC Reporter Bioassay response to cetuximab (black histograms) and rituximab (white histograms) obtained using the FcγRIIIa/NFAT-RE-luciferase expressing Jurkat cell line against P and VR CM cell lines. Results are expressed as fold induction of luminescence (Relative Light Unit, RLU) measured respectively in the presence or absence of antibodies. *p≤0.05.

Since inhibition of EGFR-driven signaling did not appear mandatory for maintaining BRAFi resistance in our model, we hypothesized that the frequent EGFR expression on CM cells acquiring BRAFi-resistance may still represent a valuable therapeutic target. Along this line, the ability of antibodies to mediate ADCC is a particularly attractive feature that led us to investigate whether the anti-EGFR antibody cetuximab could target EGFR-expressing VR cells through immune-mediated mechanisms. The ability of cetuximab to kill CM cells by triggering ADCC was first investigated using peripheral blood mononuclear cells (PBMCs) from healthy donors. As shown in **Figure 5A**, cetuximab significantly induced (p<0.05) killing of EGFR-expressing VR cells by ADCC at all effector:target ratios examined. Cell lysis ranged from 44% to 48% in Mel 611 VR, and from 22% to 29% in Mel 767 VR, at effector: target ratios of 20:1 to 80:1, respectively. The extent of cell lysis correlated with the level of cell surface expression of EGFR (**Figures 2A, B**), being higher in Mel 611 VR as compared to Mel 767 VR, and to Mel 599 VR (**Supplementary Figure 5**). Triggering of ADCC by cetuximab was specifically dependent on EGFR expression on VR cells. Indeed, specific lysis due to ADCC was not observed towards EGFR-negative Mel 611 P and Mel 767 P cell cultures, nor towards EGFR-negative P or VR Mel 919 cells. The specificity of these observations was further confirmed by the inability of the anti-CD20 antibody rituximab to mediate ADCC against any of the CM cell cultures investigated, ruling out potential non-specific activities of the added antibodies (**Figure 5A**).

It is well known that CM can promote a vast remodeling of host’s immune system, whose activity is frequently skewed towards an immunosuppressive and tumor-tolerating state within tumor microenvironment. To provide supportive evidence that the above reported observations could be potentially effective also *in vivo* in CM patients, we evaluated whether, in the presence of cetuximab, also PBMCs obtained from allogeneic metastatic CM patients were able to effectively kill *ex vivo* EGFR-positive VR CM cells through ADCC. As shown in **Figure 5B**, cetuximab significantly (p<0.05) mediated ADCC of Mel 611 VR by PBMCs from 3 metastatic CM patients at effector: target ratios of 40:1 to 80:1, with a maximum lysis of 30%, confirming that PBMCs from CM patients retained the ability to deliver cetuximab-triggered ADCC.

The ability of cetuximab to trigger ADCC towards EGFR-positive VR CM cells was further detailed by using an independent test, which evaluates the capability of antibody-cell combinations to activate the signaling cascade required for NK cell activation and target cell killing (**Figure 5C**). As expected, cetuximab was able to induce a significant (p<0.001) activation of “artificial” NK cells only when incubated with EGFR-positive Mel 611 VR, Mel 767 VR and Mel 599 VR cells, but not with EGFR-negative Mel 611 P, Mel 767 P or Mel 911 P or VR cells. No activation of the effector cells was observed following the addition of the anti-CD20 antibody rituximab, used as negative control (**Figure 5C**, **Supplementary Figure 5**).

### 2.5 Therapeutic efficacy of cetuximab treatment of BRAFi resistant melanoma cells in humanized mice

**Figure 6:**
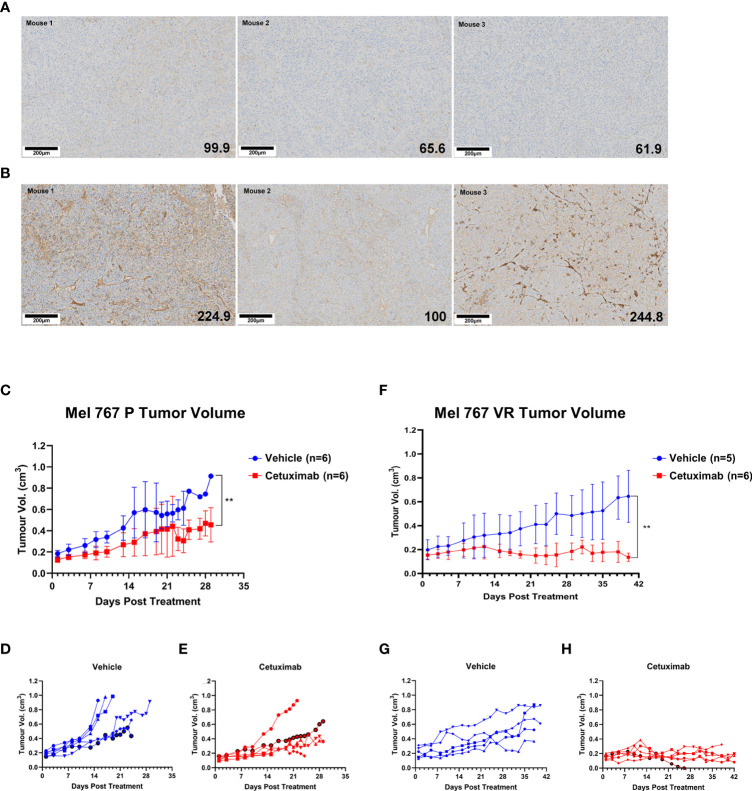
Antitumor activity of cetuximab in humanized mice. **(A, B)** EGFR IHC analysis of Mel 767 xenograft tumors. Tumor tissues harvested at ethical endpoints from NSG-SGM3 mice bearing Mel 767 P **(A)** and Mel 767 VR **(B)** were fixed, sectioned and stained for EGFR (31G7). Bottom right number indicating EGFR H-score. Tissue sections were analyzed and scored using QuPath digital pathology program. Scale bar represents 100 µm. **(C)** Mel 767 P *in vivo* xenograft tumor growth curves in humanized mice with (red) or without (blue) bi-weekly cetuximab therapy. Data are shown as mean Mel 767 P tumor volume of each treatment arm over time (days post treatment), n=6 per treatment arm. Error bars represent SD. Kruskal-Wallis test, ** p=0.0048. **(D)** Individual tumor growth curves of humanized mice bearing Mel 767 P xenograft (vehicle control). **(E)** Individual tumor growth curves of humanized mice bearing Mel 767 P xenografts treated with cetuximab. **(F)** Mel 767 VR *in vivo* xenograft tumor growth curves in humanized mice with (red) or without (blue) bi-weekly cetuximab therapy. All mice were treated with PLX4032 every two days. Data are shown as mean Mel 767 VR tumor volume of each treatment arm over time (days post treatment), n=5-6 per treatment arm. Error bars represent SD. Kruskal-Wallis test, ** p=0.0015. **(G)** Individual tumor growth curves of humanized mice bearing Mel 767 VR xenograft (vehicle control). **(H)** Individual tumor growth curves of humanized mice bearing Mel 767 VR xenografts treated with cetuximab.

Preliminary experiments allowed the identification of Mel 767 P and VR as a suitable model for vemurafenib resistant CM in mice based on their ability to form tumors *in vivo*, although the Mel 767 VR cells showed a slower growth rate, as previously shown for BRAFi-resistant cell lines (20). To test the ADCC-mediated therapeutic effect of cetuximab *in vivo*, we have set up a humanized mouse system based on the engraftment of human donor PBMCs in NSG-SGM3 mice. This mouse strain was chosen due to the expression of human stem cell factor, human Granulocyte/Macrophage‐colony stimulating factor 2 and interleukin 3 as transgenes, which allow superior engraftment of human myeloid, B cells and T cells (67, 68). To confirm that the Mel 767 VR tumors retained the same EGFR up-regulation observed in the *in vitro* cultured cell line, Mel 767 P and VR tumors grown in NSG-SGM3 mice were harvested at ethical endpoint and investigated for EGFR expression via immunohistochemistry. As shown in **Figures 6A, B**, Mel 767 VR tumors retained an up-regulated EGFR phenotype with a mean tumor H‐score of 189.9/300 respect to Mel 767 P tumors with a mean tumor H‐score of 75.8/300. It is noted that Mel 767 parental cells were not completely EGFR-negative when grown *in vivo* as compared with the same cells grown *in vitro* and investigated for EGFR expression by Western blotting.

We first investigated the efficacy of cetuximab in NSG‐SGM3 mice humanized with freshly isolated human PBMCs (10 x 10^6^ cells/mice), supplemented with IL‐15/IL‐15RαFc engrafted with EGFR‐low, vemurafenib sensitive MEL767 P cells. Based on earlier pilot tumor grow curve studies, mice were injected with 2.5 x 10^6^ Mel 767 P cells with Matrigel (1:1 ratio). Once Mel 767 P tumors were established and grown to a palpable size of ~0.2cm^3^, mice were bi‐weekly i.v. injected with cetuximab (400 μg/mouse) for 3 weeks or treated with vehicle (saline) (n=6/group). As shown in **Figures 6C–E**, tumor growth in mice treated with cetuximab was slightly, although significantly (p = 0.0048), inhibited compared to the control group, with only 1/6 treated mouse undergoing faster tumor progression.

To investigate the *in vivo* efficacy of cetuximab against VR cells, NSG‐SGM3 mice were injected with 3.5 x 10^6^ Mel 767 VR cells with Matrigel (1:1 ratio) and treated with 10 mg/kg vemurafenib every 2 days to ensure that these tumors maintained also *in vivo* their vemurafenib‐resistant phenotype along with EGFR up-regulation. As for the experiment with vemurafenib sensitive cells, NSG‐SGM3 mice were humanized with freshly isolated human PBMCs (10 x 10^6^/mice), supplemented with IL‐15/IL‐15RαFc when Mel 767 VR tumors were established and grown to a palpable size of ~0.2cm^3^ and treated with cetuximab or vehicle (n=5-6/group). As shown in **Figures 6F–H**, cetuximab induced a strong and significant inhibition of Mel 767 VR tumors (p < 0.008) whose growth was stabilized in all cases except for one mouse which had tumor clearance but had to be culled early due to early onset of graft vs. host disease (GvHD). By contrast, all animals treated with the vehicle (5/5) (**Figures 6F, G**) developed tumors showing sustained growth, consistently with their VR phenotype. These findings indicate that cetuximab may be an effective therapeutic option to control BRAFi-resistant CM.

## 3 Discussion

In the present study, we provide evidence indicating that targeting a shared phenotype imposed by BRAFi resistance constitutes an effective modality to kill BRAFi-resistant cells both *in vitro* and *in vivo*, independently of the underlying resistance mechanism. In particular, we confirmed that gain of EGFR expression is a common phenotypic trait of CM cells acquiring resistance to BRAFi, and demonstrated that the anti-EGFR, ADCC-mediating, mAb cetuximab can effectively kill EGFR-positive BRAFi resistant CM cells through an immune-mediated mechanism.

Among the BRAFi-resistant cell lines generated in our study, 3 out of 9 (33%) showed *de novo* cell surface expression of EGFR, while 1 proved EGFR-positive both in the VR and in the respective P BRAFi-sensitive cultures. Even though the number of cell lines used in this study is limited, the prevalence of *de novo* EGFR expression in VR cultures is in line with those reported for tissues from a study by Sun C et al. (20). Subsequently, Ji Z et al. reported that 4/5 CM tumor pairs from CM patients treated with BRAF ± MEK inhibitors showed increased EGFR expression in the post-relapse samples compared to pre-treatment samples (16). Similarly, Wang J. et al. compared EGFR expression in autologous pairs of CM specimens obtained from 12 different patients before and after treatment with BRAFi, and EGFR expression post-treatment was significantly higher in almost all BRAFi resistant recurrent tumors (30). In a more recent study, EGFR mRNA levels could be measured in 3/5 CM patients who relapsed after 3-15 months from the end of the treatment with vemurafenib (69). Taken together, these data suggest that therapeutic approaches based on anti-EGFR ADCC-mediating mAbs could be applicable in at least 30% of BRAFi relapsing patients. Besides, immunohistochemistry performed on tumor samples from 19 patients showed EGFR tumor positivity in pre-therapy lesions in 16% of patients (**Supplementary Table 4**), suggesting its potential targeting independently from BRAFi-treatment or in CM that are intrinsically resistant to BRAFi and that are marked by EGFR upregulation (36). The potential clinical utility of immune targeting EGFR-positive CM cells even before or concomitantly to targeted therapy could find additional rationale from a recent paper showing the presence of rare cell populations variants, marked by surface EGFR-expression, that are poised to resistance to targeted therapies and which could contribute to tumor outgrowth upon BRAF/MEK inhibition (70). Unfortunately, post-BRAFi lesions were not sufficiently represented in the patient cohort that we had available to allow us to reliably assess the prevalence of EGFR expression on tumor tissues following gain of BRAFi-resistance.

ADCC can be mediated by both NK cells, mainly activated by IgG1 isotype antibodies, and myeloid-derived effectors, triggered by both IgG1 and IgG2 antibodies (71). In our model, anti-EGFR mAb-triggered ADCC of VR cells was essentially delivered by NK cells since targeting EGFR by the IgG2 isotype mAb panitumumab did not result in a significant killing of EGFR-positive VR cells (data not shown), suggesting a negligible, if any, contribution of myeloid-derived effectors in mAb mediated killing in this setting. In line with this finding, IgG1 class anti-EGFR mAbs (e.g., cetuximab, nimtuzumab, and necitumumab) appear preferable for combined therapies targeting EGFR-positive BRAFi-relapsing CM. A further improvement in this respect could be achieved by using anti-EGFR antibodies optimized to deliver enhanced ADCC activity, which demonstrated a superior anti-tumor activity as compared to cetuximab in mouse models (72).

As shown by Ji Z et al., CM lineage reprogramming contributes to BRAFi resistance through induction of autocrine EGFR ligand expression that triggers EGFR signaling (16). Engagement of this autocrine loops is potentially shared by other RTKs expressed following establishment of BRAFi-resistance, as demonstrated by the coordinate *de novo* expression of AXL and PDGFRβ (**Supplementary Figure 6**, **Supplementary Table 5**) and their ligands (i.e. GAS6 and PDGF) in our VR cell lines (**Figures 1A, B**). Accordingly, we confirmed that the EGFR-ligand NRG1 was consistently co-expressed with EGFR in VR cell cultures (**Figure 1H**). Since cetuximab binds EGFR at the ligand binding site, autocrine ligand production could be able to interfere with the binding/activity of the mAb. Though we did not specifically address this issue from a mechanistic point of view, our data suggest that autocrine EGFR ligand expression did not significantly prevent the binding of cetuximab, nor the killing of VR CM cells by ADCC, supporting the efficacy of targeting RTKs through immune-mediated mechanisms even in the presence of autocrine ligand production.

From a perspective therapeutic point of view, a major advantage of using anti-EGFR (or more in general anti-RTK) ADCC-mediating mAbs is their ability to kill RTK-expressing VR CM cells independently of the requirement of downstream signaling for sustaining resistance to BRAFi, as it has already been demonstrated in other contexts (51, 73). Indeed, in our model, we found that cetuximab is at least as effective as gefitinib in down-modulating EGFR phosphorylation in VR cells, either it being constitutive or following ectopic EGF, in a cell context-dependent manner. Nevertheless, we failed to demonstrate a significant impact on BRAFi sensitivity of EGFR inhibition through either small molecule inhibitors or cetuximab. In addition, complete knock-down of EGFR protein expression in Mel 611 VR cells by genomic editing approaches confirmed that, at least in this specific cell line, EGFR expression is unnecessary for maintaining BRAFi-resistance. These observations are in line with reports suggesting that BRAFi resistance may result from a deep reprogramming of cellular signaling triggered by a switch to a different cellular state. In this context, the alternative cellular state was consistently marked by specific RTK profiles, but resistance was independent of the activity of distinct RTKs (20, 24, 36). Accordingly, inhibition of single RTK (e.g., EGFR, AXL) did not result in markedly restored sensitivity to BRAFi, as it did, in contrast, the targeting of common downstream signaling nodes/mediators (e.g. AKT, SRC) or multiple RTK targeting (20, 24, 29, 36, 74, 75).

Our data do not completely exclude that single RTK could play a prominent role in maintaining BRAFi resistance (19, 29, 30, 33, 38), nonetheless they clearly indicate that EGFR expression is dispensable for maintaining resistance to PLX4032 in specific CM cells. In these experimental conditions, the ability of cetuximab to mediate ADCC effectively killed EGFR-positive VR cells, suggesting that the use of ADCC-mediating anti-RTK mAbs could prove therapeutically valid, thanks to their immune-mediated mechanisms of action, independently of their effect on signaling. These therapeutic properties could further benefit from the ability of cetuximab-activated NK cells to induce an adaptive T-cell response against tumor antigens expressed by EGFR-positive cancer cells (52), which could boost the adaptive immune response to EGFR-expressing VR cells.

Our observations are likely not limited to EGFR but could prove valid also for other RTKs that are *de novo* expressed on VR cells. Among these, AXL is particularly appealing since it is included in the RTK signatures of BRAFi resistant CM (**Figure 1A**), and it has been proposed to play a potentially important role in acquiring resistance to BRAFi and in increasing the invasive potential of CM cells (24, 33, 76–78). Noteworthy, a recent single cell sequencing study revealed that a population resistant to targeted-therapy labeled as “invasive” and expressing high levels of AXL became frequently expanded in CM patient derived xenografts progressing on combined BRAF/MEK/RXR inhibition (18). We confirmed AXL to be *de novo* expressed on the cell surface of 2 out of the 9 VR CM cultures analyzed (**Supplementary Figure 7**). As seen with EGFR, AXL expression appeared to be dispensable for maintaining resistance to BRAFi, since its genetic knock down did not restore CM sensitivity to vemurafenib (**Supplementary Figure 8**). Independently on its role on BRAFi-resistance, AXL expression on VR cells makes it an attractive target for the ADCC-mediating anti-AXL mAbs that are currently being developed (79, 80). However, whether the expression of AXL on both tumor-associated and normal endothelial cells (**Supplementary Table 4**) could lead to systemic toxicities of anti-AXL mAbs has to be considered and explored.

In consideration of their ability to favor the cross-talk between innate and adaptive immune responses, an intriguing therapeutic option for the clinical use of ADCC mediating anti-RTK antibodies in BRAFi-relapsing CM is the combination with currently used immune checkpoint inhibitors (ICIs) that demonstrated to provide long term clinical control of the disease in this malignancy (81–83). Moreover, CM cells acquiring BRAFi resistance through activation of alternative signaling pathways driven by *de novo* expressed RTK(s) were shown to up-regulate the cell surface expression of PD-L1 molecule. This molecular event could further contribute to the progression of BRAFi resistant CM by favoring their immune escape mediated by triggering of the inhibitory PD1 immune checkpoint in tumor infiltrating effector T cells (84–86). Of interest, RNA sequencing analysis revealed that the expression of PDCD1LG2, also known as PD-L2, was significantly increased following acquisition of BRAFi resistance (**Supplementary Figure 9**). PD-L2 has been described as a second ligand for PD-1 in addition to PD-L1 (87), thus representing a potential target for cancer immunotherapy with PD-1 inhibitors. Hence, in this context, targeting of EGFR-positive BRAFi-resistant CM cells by combined treatment with cetuximab and ICIs might appear an extremely attractive option that would take advantage of the concomitant action on tumor and immune cells.

Our results obtained in humanized mice are consistent with the observation that cetuximab may exert some degree of therapeutic efficacy *in vivo* against BRAFi-responsive melanoma cells favored by a low basal level of EGFR expression. More important in a clinical perspective is our finding that cetuximab strongly inhibited the growth of a VR melanoma cell line in humanized mice undergoing concomitant vemurafenib treatment. Considering the emerging relevance of humanized mice as fundamental preclinical platforms for drug testing and therapeutic screening, as recently stated by the FDA (88), these findings provide the proof-of-principle to activate clinical trials aiming at assessing the therapeutic efficacy of cetuximab to improve the control of BRAFi in CM. In this context, it will be noteworthy to first evaluate the potential side effects of cetuximab therapy on EGFR-expressing normal epithelial cells. Several studies reported frequent dermatological toxicities of EGFR-targeting agents due to the key function of EGFR in skin biology (89). Acneiform eruptions were reported in patients affected by lung, colorectal, or head and neck cancers and treated with EGFR-inhibitors. However, severe skin toxicities could be well prevented by oral antibiotics administration (90). Interestingly, cetuximab treatment in locally advanced cutaneous squamous cell cancer was associated with only mild acneiform skin rash, thus suggesting the potential safety profile of this drug in cutaneous malignancies (91).

In conclusion, our study, conducted on a wide panel of P and VR pairs of CM cells, confirmed a frequent *de novo* expression of RTKs in CM cells acquiring BRAFi resistance, and demonstrated that their targeting by ADCC-triggering mAbs can efficiently and reliably kill RTK(s)-expressing VR CM cells and mediate therapeutic effects *in vivo*. These data provide the grounds for the evaluation of the therapeutic activity of anti-RTK mAbs in CM relapsing on BRAFi, where combinations with immune checkpoint inhibitors appear a particularly suited setting.

## 4 Materials and methods

### 4.1 Cells cultures and reagents

Cell cultures were established from metastatic lesions surgically removed from cutaneous CM patients that did not underwent prior BRAFi therapy who were referred to the Centro di Riferimento Oncologico, IRCCS-National Cancer Institute, Aviano, Italy, as previously described (92). VR cell cultures were generated from BRAF V600 mutant, PLX4032-sensitive, CM cells by sequential culturing in the presence of doubling concentrations of PLX4032 (Selleck Chemicals), starting from 0.25 µM until they were able to grow in 8 µM PLX4032. Cell cultures were grown in RPMI 1640 Medium, supplemented with 2mM L-glutamine (Sigma-Aldrich) and 10% heat-inactivated fetal calf serum (FCS, Lonza). VR cell cultures were propagated under the same culture conditions, with the addition of 8 µM PLX4032. The identity of paired P and VR cells was confirmed by short tandem repeat profiling using the Power Plex 1.2 kit (Promega). Mutation status in BRAF and NRAS genes was determined as previously described (93, 94). PBMCs from healthy donors and CM patients were prepared as previously described (95). The study was approved by the Internal Review Board of the Centro di Riferimento Oncologico, IRCCS-National Cancer Institute, Aviano, Italy (IRB number 07-2017). Stock solutions of gefitinib and PLX-4032 (SeleckBio) were prepared in cell-culture grade DMSO (Sigma) as per manufacturer’s indications. Cetuximab (MERK) and EGF (Life Technologies) stocks were diluted in RPMI medium.

### 4.2 RNA sequencing

Libraries preparation was performed as described in Montico et al. (96). RNA purity and integrity were assessed with a Nanodrop 2000c spectrophotometer (Thermo Fisher Scientific, Waltham, MA, USA) and a 4200 TapeStation instrument (Agilent Technologies, Santa Clara, CA, USA), respectively. For RNA purity, an A260/280 ratio of ˜2.0 and an A260/230 ratio of 2.0-2.2 were considered acceptable; for RNA integrity, an RNA Integrity Number (RIN) of 9.0-10.0 has been obtained for all samples, indicating the absence of degradation and high integrity of RNA samples. For a precise estimation of the RNA concentration, a Qubit 2.0 fluorometer assay (Thermo Fisher Scientific, Waltham, MA, USA) has been employed. For RNA sequencing, 1 μg of high-quality total RNA was used for library preparation with a TruSeq Stranded Total RNA Sample Prep Kit (Illumina, San Diego, CA, USA) and sequenced (paired-end, 2 x 75 cycles) on the NextSeq 500 platform (Illumina, San Diego, CA, USA). For each experimental condition, three biological replicates were considered. RNA sequencing data analysis was performed as described in Casarotto et al. (97). In detail, the raw sequence files generated (.fastq files) underwent quality control analysis using FASTQC (http://www.bioinformatics.babraham.ac.uk/projects/fastqc/) and adapter sequences were removed using Trimmomatic version 0.38 (98). Filtered reads were aligned on human genome (assembly hg38) considering genes present in GenCode Release 37 (GRCh38.p13) using STAR v2.7.9a (99) with standard parameters. Quantification of expressed genes was performed using featureCounts (100) and differentially expressed genes were identified using DESeq2 (101). A given RNA was considered expressed when detected by at least ≥ 10 raw reads. Differential expression was reported as |fold-change| (FC) ≥1.5 along with associated adjusted p-value ≤ 0.05 computed according to Benjamini-Hochberg. Functional analysis on differentially expressed genes was performed using Ingenuity Pathway Analysis (IPA, Qiagen). Only Canonical Pathways and Molecular Function with a p-value < 0.05 were considered for further analysis. Prediction of Transcriptional regulators of the differentially expressed genes was performed using ChEA3 (102). The tables with the normalized values of expressed transcripts are available in BioStudies repository (https://www.ebi.ac.uk/biostudies) with the accession number S-BSST1225. The raw data of the RNA sequencing datasets generated during and/or analyzed during the current study are available from the corresponding author on reasonable request.

### 4.3 Quantitative RT-PCR analysis

Real-time quantitative PCR analyses were performed as described (103) using Power SYBR Green Master Mix (Life technologies). Primers sets used are listed in **Supplementary Table 6**. The absolute copy number of cDNA of target genes and of the reference gene β-actin were measured in each sample from standard curves. The number of target gene cDNA molecules in each sample was normalized to the number of cDNA molecules of β-actin. The Student’s t-test for two tailed distributions and paired data was used to compare normalized gene expression between VR and P CM cell lines. Differences were considered statistically significant when p≤ 0.05.

### 4.4 Dose-response curves

CM cells were seeded in flat bottom 96-well plates at a density of 2500 or 5000 cells/well, depending on their growth rate. After 24h, scalar doses of PLX-4032, or an equal volume of DMSO, used as negative control, were added into triplicate wells. When combination experiments were performed, fixed concentrations of gefitinib (2.5 µM), cetuximab (20 µg/ml) and/or EGF (20 ng/ml) were added to both DMSO-treated and PLX-4032-treated wells. Cell viability was evaluated 72h after the addition of PLX-4032 by a standard MTT assay (Life Technologies). Generation of sigmoidal dose-response curves using a four-parameter nonlinear regression model, and calculation of PLX-4032 IC_50_ values were achieved by the Prism 6.0 software (GraphPad Software).

### 4.5 Flow cytometry analysis

Flow cytometry analyses were performed essentially as previously described (104). EGFR was detected by the phycoerythrin-conjugated anti-EGFR clone EGFR.1 mouse IgG2b mAb (Becton Dickinson); a phycoerythrin-conjugated mouse IgG2b isotypic antibody (Becton Dickinson) served as negative control. Antibodies were used following the manufacturer’s instructions. Data acquisition was performed with a FACSCanto II flow cytometer (Becton Dickinson) and analyzed with Diva 5.0 (Becton Dickinson) and FlowJo software (Tree Star, Inc).

### 4.6 Western blot analysis

CM cells were plated in 6-well plates. Twenty-four hours after plating, cells were added with DMSO or 20 µg/ml cetuximab, 2.5 µM gefitinib, with or without 20 ng EGF, and were analyzed following 24h incubation. Whole cell lysate preparation and western blotting were performed as previously described (104). Ten µg cell lysate were separated on Criterion TGX 4-15% gradient gels (BioRad) and blotted onto nitrocellulose membranes. Immunoblotting was performed using the following antibodies, under the manufacturer’s instructions: anti-phospho-Akt (Ser473) (#9271), -total-AKT (#9271), -phospho-EGFR (Tyr1068) (#3777), and -total-EGFR (#2232) antibodies from Cell Signaling Technology; anti-β-tubulin (H-235) (sc-9104), used as a loading control, from Santa Cruz Biotechnology. Revelation was performed using the Clarity Western ECL Substrate (BioRad) or the SuperSignal Femto reagent (Pierce) through Chemidoc XRS+ instrument (Biorad). Image analysis was performed with the Image Lab v6.1 (Biorad) software. The Student’s t-test for two tailed distributions was used to compare data. Differences were considered statistically significant when p≤ 0.05.

### 4.7 CRISPR/Cas9 genomic editing

Guide sequences targeting EGFR were either previously described [TGCAAATAAAACCGGACTGA, exon 3 (105)] or designed (TCCTCCAGAGCCCGACTCGC, exon 1) using the CRISPR design software available at http://crispr.mit.edu. Complementary oligonucleotides containing cloning overhangs were synthesized at Sigma, annealed, and the obtained double stranded oligonucleotide was cloned into the pSpCas9(BB)-2A-GFP (PX458) plasmid, kind gift from Feng Zhang (Addgene plasmid # 48138), as per inventor’s protocol (106). Plasmids were then transfected into CM cells using Lipofectamine 2000 reagent (Life Technologies) following the manufacturer’s instructions. EGFR-negative VR CM cells were sorted 1 week after transfection using FacsARIA III (Beckton Dickinson).

### 4.8 *In vitro* assays to quantify ADCC

The ability of cetuximab to mediate ADCC in EGFR-expressing CM cell lines was evaluated through 2 different *in vitro* assays: a flow cytometry-based assay [modified from Hermans et al. (107)] and the ADCC Reporter Bioassay (Promega). In the flow cytometry approach, P and VR CM cell lines were labeled with 2 different concentrations (0.3125µM and 3.75µM respectively) of Carboxyfluorescein succinimidyl ester (Vybrant^®^ CFDA SE cell Tracer, CFSE; Molecular Probes, Life Technologies) for 10 minutes in 500 µl of PBS; FCS (Lonza) was added for 20 minutes to quench the reaction, and cells were washed 4 times. VR and P CFSE-labeled cells were then counted and incubated together at a concentration of 2x10^5^/ml in Hank’s Balance Salt Solution (Sigma) added with 10% FCS (Lonza). So treated target cells were then co-cultured overnight together with PBMCs obtained from healthy donors or CM patients, at different effector:target ratios (80:1; 40:1; 20:1; 0:1), in the presence/absence of 20 µg/ml of cetuximab, using 96-well ultra-low attachment microplates (Corning) at 37°C and 5% of CO_2_. Rituximab (20 µg/ml)-labeled cells were used as negative control. Samples were collected and acquired with a FC500 flow cytometer (Beckman Coulter) and data were analyzed with the FlowJo software (Treestar). Specific lysis was calculated using Sytox^®^ Orange Dead Cell Stain (Life Technologies) as cell death marker. ADCC efficiency was expressed as adjusted lysis, calculated as 100-adjusted survival, i.e. 100*(survival with effectors/survival without effectors). The Student’s t-test for two tailed distributions and paired data was used to compare ADCC efficiency in the presence or absence of antibodies, or mediated by cetuximab versus rituximab. Differences were considered statistically significant when p≤ 0.05. The ADCC Reporter Bioassay (108) was performed under manufacturer’s instructions. Briefly, P and VR CM cell lines were incubated at a 1:1 ratio with FcγRIIIa/NFAT-RE-luciferase expressing Jurkat cell line for 6 hours at 37°C and 5% of CO_2_ in the presence/absence of cetuximab or rituximab (10 µg/ml). Luciferase activity was quantified using the Bio-GloTM Reagent and luminescence was measured using Tecan Infinite 200 Pro (Tecan Group Ltd). Data were expressed as luminescence (Relative Light Unit, RLU) fold of induction = RLU induced (cetuximab or rituximab)/RLU no antibody control. The Student’s t-test for two tailed distributions and paired data was used to compare RLU fold induction in the presence of cetuximab or rituximab, or induced by cetuximab/rituximab in VR or P CM cell lines. Differences were considered statistically significant when p≤ 0.05.

### 4.9 *In vivo* efficacy studies

NOD.Cg-Prkdcscid Il2rgtm1Wjl Tg(CMV-IL3,CSF2,KITLG)1Eav/MloySzJ (NSG-SGM3) mice between 6-8 weeks of age of mixed gender were obtained from Prof. Kristen Radford’s research group (Mater Medical Research) originally sourced from JAX laboratories (Maine, USA). Animal experiments were approved by the University of Queensland Animal Ethics Committee (AEC 060/19). For tumor cell engraftment, Mel 767 P and VR cells were resuspended in 1:1 PBS : Matrigel (corning) suspension in a cell density of 2.5 x 10^7^ and 3.5 x 10^7^ cells/mL respectively and kept on ice till injection. Mice were anesthetized with 2-3% isoflurane and 100 µL of cell suspensions (Mel 767 P: 2.5 x 10^6^ cells/mice, Mel 767 VR 3.5 x 10^6^ cells/mice) were injected subcutaneously into the right or left flank. Mice bearing Mel 767 VR tumor cells were treated with 10 mg/kg vemurafenib (PLX4032) (MedChemExpress) intraperitonially (i.p) every 2 days. When tumors reach 0.2-0.3 cm^3^ in size, tumor bearing NSG-SGM3 mice were humanized with freshly isolated human PBMC from healthy donors (human ethics HREC/2018/QMS/44046). 10 x 10^6^ human PBMCs in 100 µL volume of PBS was injected via tail vein intravenous (i.v) injection. After human PBMC injection, mice were monitored and scored for signs of GvHD. Submandibular blood collection of humanized animals for immune analysis was performed two weeks post human PBMC injection to confirm successful engraftment of human immune cells. For maintenance of NK cells post PBMC engraftment, mice were injected i.p with 2.5 µg of recombinant human IL-15 (Peprotech) complexed with recombinant Human IL-15Rα Fc Chimera (R&D systems) (huIL-15/IL-15RαFc) once weekly. Cetuximab treatment commenced immediately a day after humanization and 400 µg/mice of cetuximab were injected into mice bi-weekly via tail vein i.v injection for 3 weeks. In vehicle control group, mice received saline injection. Mice were scored, weighed and tumors were measured via caliper measurement every 2 days.

## Data availability statement

The datasets presented in this study can be found in online repositories. The names of the repository/repositories and accession number(s) can be found below: https://www.ebi.ac.uk/biostudies/studies/S-BSST1225?key=1e247f11-3430-47c2-bc66-ad69b16dc5d0.

## Ethics statement

The studies involving humans were approved by Internal Review Board of the Centro di Riferimento Oncologico, IRCCS-National Cancer Institute, Aviano, Italy (IRB number 07-2017). The studies were conducted in accordance with the local legislation and institutional requirements. The human samples used in this study were acquired from metastatic lesions surgically removed from cutaneous CM patients that did not underwent prior therapy with BRAF inhibitors who were referred to the National Cancer Institute of Aviano (Italy), as described in “Altomonte M et al. Differential Expression of Cell Adhesion Molecules Cd54/Cd11a and Cd58/Cd2 by Human Melanoma Cells and Functional Role in Their Interaction with Cytotoxic Cells. Cancer Res (1993) 53(14):3343-8”. Written informed consent for participation was not required from the participants or the participants’ legal guardians/next of kin in accordance with the national legislation and institutional requirements. The animal study was approved by Animal procedures were approved by the University of Queensland Animal Ethics Committee (approval number: UQDI/491/15/NHMRC/ARC). The study was conducted in accordance with the local legislation and institutional requirements. Written informed consent was obtained from the individual(s), and minor(s)’ legal guardian/next of kin, for the publication of any potentially identifiable images or data included in this article.

## Author contributions

EM: Conceptualization, Data curation, Investigation, Writing – original draft, Writing – review & editing. BM: Conceptualization, Data curation, Investigation, Writing – original draft, Writing – review & editing. BL: Data curation, Investigation, Writing – review & editing. FC: Investigation, Writing – review & editing. GG: Data curation, Investigation, Software, Writing – review & editing. AS: Writing – review & editing, Data curation, Investigation. RG: Writing – review & editing, Investigation. AR: Writing – review & editing, Investigation. EC: Writing – review & editing, Investigation. VC: Writing – review & editing, Data curation. AA: Writing – review & editing, Data curation. MD: Writing – review & editing, Data curation, Investigation. RM: Writing – review & editing, Data curation, Investigation. MM: Writing – review & editing, Data curation, Investigation. AW: Writing – review & editing, Data curation, Funding acquisition. MP: Writing – review & editing, Data curation, Funding acquisition. FS: Data curation, Funding acquisition, Investigation, Writing – review & editing. RD: Conceptualization, Data curation, Methodology, Project administration, Supervision, Writing – review & editing. EF: Conceptualization, Data curation, Funding acquisition, Investigation, Methodology, Project administration, Supervision, Writing – original draft, Writing – review & editing. LS: Conceptualization, Data curation, Funding acquisition, Investigation, Methodology, Project administration, Supervision, Writing – original draft, Writing – review & editing.
