# Supplementary material for: Antibody dependent cellular cytotoxicity-inducing anti-EGFR antibodies as effective therapeutic option for cutaneous melanoma resistant to BRAF inhibitors
Source: Front Immunol. 2024 Mar 6;15:1336566. doi: 10.3389/fimmu.2024.1336566 (PMC10950948; doi:10.3389/fimmu.2024.1336566)
Supplement: Supplementary file 1 [file DataSheet_1.pdf]

Supplementary Figure 1. PLX4032 sensitivity of P and VR CM cells. P or VR CM cells were seeded in 96 well plates and treated for 72h with scalar doses of PLX4032. Cell viability was evaluated using standard 4h MTT assays. Dose-response curves report means and standard deviations of three replicates vs. logarithmic scale of PLX4032 concentration. Normalized viability (%) is relative to vehicle-treated cells. For each isogenic CM cell pair, IC_{50} values of PLX4032 for P and VR cells are listed above each graph.

Supplementary Figure 2. Analysis of TCGA samples and pathway analysis on RNA sequencing data. Violin_plots reporting the log2(TPM+1) transcript values for RTK (A), RTK _Ligands (B) and transcription factors (C) (genes listed in Figure 1) in the TCGA SKCM dataset according to the predicted intrinsic resistance or sensitivity to BRAF inhibitors. *, $p \leq 0.05$. (D), left panel, barplots showing EGFR-related signaling pathways significantly enriched in VR vs P cells: Mel 599, blue; Mel 611, orange; Mel 767, green. On the right panel, the same analysis performed on the TCGA SKCM dataset, comparing samples predicted to be intrinsically resistant vs. sensitive to BRAF inhibitors.

Supplementary Figure 3. Densitometric analyses of EGFR signalling in P and VR cell cultures. P and VR cells were seeded in 6 well plates and treated for 24 h with either DMSO, 20 μg/ml cetuximab (CTX) or 2.5 μM gefitinib (GEF), with or without the addition of 20 ng/ml EGF. VR cells were maintained without PLX4032 for the duration of the assay. Immunoblotting was performed on cell lysates to evaluate EGFR signalling by EGFR and AKT phosphorylation (pEGFR, pAKT). Total EGFR and AKT (EGFR, AKT) served as reference, β-tubulin served as loading control. Quantifications represent the densitometry values of the protein normalized to those of β-tubulin and referred to VR DMSO set to 1. Data from 3 independent experiments. Comparisons evaluated: pEGFR: VR DMSO vs. VR EGF; VR EGF vs. VR EGF+CTX; VR EGF vs. VR EGF+GEF; EGFR: P DMSO vs. VR DMSO; VR DMSO vs. VR EGF; pAKT: P DMSO vs VR DMSO; VR DMSO vs. VR EGF; VR EGF vs VR EGF+CTX; VR EGF vs VR EGF+GEF. *, $p<0.05$, Student's T test.

Supplementary Figure 4. Effect of EGFR-inhibition on colony forming ability of P and VR cells under PLX-4032 treatment. P and VR cells were seeded in 6-well plates. After 24h, cells were added with DMSO (negative control), 2.5 µM gefitinib (GEF 2.5), 5 µM gefitinib (GEF 5), or 20 µg/ml cetuximab (CTX), with or without 4 µM PLX-4032. Colonies were evaluated after 2 weeks from plating.

Supplementary Figure 5. Antibody-Dependent Cell Cytotoxicity (ADCC) mediated by EGFR- and CD20-targeting antibodies. (A) Representative graphs of ADCC mediated by cetuximab (black square) in Mel 599 P and Mel 599 VR cell lines. Data are shown as mean and standard deviation of 3 independent experiments performed with PBMCs obtained from 3 different healthy donors at 4 different effector:target ratios (80:1, 40:1, 20:1, 0:1), co-cultured overnight with target cells at 37°C and 5% of CO2. ADCC efficiency is expressed as adjusted lysis, calculated as 100-adjusted survival, i.e. 100*(survival with effectors/survival without effectors). The specific lysis values measured in the presence of rituximab (black triangle) and in the absence of antibodies (white circle) are considered as negative controls. The X axis reported the different effector:target (E:T) ratios used. (B) ADCC Reporter Bioassay response to cetuximab (black histograms) and rituximab (white histograms) obtained using the FcgRIIIa/NFAT-RE-luciferase expressing Jurkat cell line against Mel 599 P and Mel 599 VR cell lines. Results are expressed as fold induction of luminescence (Relative Light Unit, RLU) measured respectively in the presence or absence of antibodies. (C) ADCC Reporter Bioassay response to cetuximab (black histograms), rituximab (white histograms), and an anti-CD20 control antibody (grey histograms) obtained using the FcgRIIIa/NFAT-RE-luciferase expressing Jurkat cell line against the Raji cell line. Results are

expressed as fold induction of luminescence (Relative Light Unit, RLU) measured respectively in the presence or absence of antibodies. *$p \leq 0.05$.

Supplementary Figure 6. AXL and PDGFRβ transcripts analysis in P and VR CM cells. Total RNA was extracted from P and VR cell cultures, retro-transcribed and subjected to SYBR Green quantitative real-time PCR analysis using assays for transcripts encoding AXL and PDGFRβ, as well as the housekeeping gene β-actin. Level of gene expression is reported as number of molecules of the target gene normalized to the number of β-actin molecules. Data are presented as mean + standard deviation of values obtained from at least 3 independent experiments; * $p \leq 0.05$.

Supplementary Figure 7. Cell surface expression of AXL on P and VR CM cells. The cell-surface expression of AXL was evaluated on P and VR isogenic cell cultures by flow cytometry. Filled gray histograms refer to isotype labeled cells; black empty histograms refer to cells labeled with anti-EGFR antibody.

Supplementary Figure 8. Effect of AXL knock down by genomic editing on PLX4032 sensitivity. The expression of AXL protein in Mel 611 VR cells was knocked down by CRISPR-CAS9. AXL-negative genome-edited Mel 611 VR cells were viably sorted by FACS and used for further assays. **A**, flow cytometry analysis confirming absence of cell surface AXL expression on Mel 611 VR CRISPR-edited (blue line). **B**, dose-response curves to PLX4032 of Mel 611 P, VR and VR CRISPR-edited at AXL (Mel 611 VR - CRISPR AXL). Cells were seeded in 96 well plates and treated for 72h with scalar doses of PLX4032. Cell viability was evaluated using standard 4h MTT assays. Dose-response curves report means and standard deviations of three replicates vs. logarithmic scale of PLX4032 concentration. Normalized viability (%) is relative to vehicle-treated cells.

Supplementary Figure 9. Heatmap showing the differentially expressed transcripts involved in the pathway "PD-1, PD-L1 cancer immunotherapy pathway" that have been found statistically significant in the three datasets (Mel 599 VR vs P, Mel 611 VR vs P and Mel 767 VR vs P) with Ingenuity Pathway Analysis.
