# Supplementary material for: Antibody dependent cellular cytotoxicity-inducing anti-EGFR antibodies as effective therapeutic option for cutaneous melanoma resistant to BRAF inhibitors
Source: Front Immunol. 2024 Mar 6;15:1336566. doi: 10.3389/fimmu.2024.1336566 (PMC10950948; doi:10.3389/fimmu.2024.1336566)
Supplement: Supplementary file 2 [file DataSheet_2.pdf]

BRAF and NRAS status
The mutational status of BRAF V600 (exon 15) and NRAS Q61 (exon 3) was determined in CM cell lines with Sanger sequencing as previously described (1, 2). Primers sets used are listed in Supplemental Table 3.

Colony forming assay
Cells were seeded in 6-well plates at a density of 30.000 cells/well. After 24h, cells were added with DMSO (negative control), 2.5 µM gefitinib, 5 µM gefitinib, or 20 µg/ml cetuximab, with or without 4 µM PLX-4032. Complete medium with drugs was substituted every 3-4 days, and colonies were evaluated after 2 weeks from plating. Colony imaging was performed by a Chemidoc XRS+ imaging system and QuantityOne software (Bio Rad) after a 20-minute staining with a 0.05% crystal violet, 1% formaldehyde, 1% methanol solution.

Immunohistochemistry analysis of EGFR and AXL expression on melanoma tissues
Immunohistochemistry was performed as described (3) on formalin-fixed, paraffin-embedded (FFPE) tissues from pre- or post-therapy lesions surgically removed from advanced melanoma patients treated with the BRAFV600E inhibitor Vemurafenib. Sections were stained with antibodies to AXL (AF154, R&D Systems) and to EGFR (4267, Cell Signaling). Sections were evaluated by a semi quantitative scoring system (4). Each antigen was assessed for expression on neoplastic cells, or on intra-tumor stromal cells, or on extra-tumor stromal cells. For each marker the expression (E) was ranked from 1 to 4 as follows: 1: immunoreactivity in up to 25% cells; 2: expression in 26–50% cells; 3 expression in 51–75% cells: 4 expression in >76–100% cells. Immunostaining intensity (I) was ranked as low (1; fainter than internal controls), normal (2; as faint as controls), or strong (3; more intense than controls). E and I were combined into a single score (S), calculated as E×I.

Flow cytometry analysis
Flow cytometry analyses were performed essentially as previously described (5). AXL was detected by the Alexa-fluor488-conjugated anti-AXL Monoclonal Mouse IgG1(R&D sistems); a Mouse IgG1/FITC, Clone DAK-GO1 isotypic antibody (DAKO) served as negative control. Antibodies were used following the manufacturer's instructions. Data acquisition was performed with a FACSCanto II flow cytometer (Becton Dickinson), and analyzed with Diva 5.0 (Becton Dickinson) and FlowJo software (Tree Star, Inc).

CRISPR/Cas9 genomic editing
Guide sequences targeting AXL [(CCATAACGCCAAGGGGGTCACCA, exon 3), (CCTGAGCGGCATCTACCCCCTGA, exon 6), (ACATTAGTGCTACGCGGAATGGG, exon 8)] were designed using the CRISPR design software available at http://crispr.mit.edu. Complementary oligonucleotides containing cloning overhangs were synthesized at Sigma, annealed and the obtained double stranded oligonucleotide was cloned into the pSpCas9(BB)-2A-GFP (PX458) plasmid, kind gift from Feng Zhang (Addgene plasmid # 48138), as per inventor's protocol (6). Pasmids were then transfected into CM cells using Lipofectamine 2000 reagent (Life Technologies) following the manufacturer's instructions. AXL-negative VR CM cells were sorted 1 week after transfection using FacsARIA III (Beckton Dickinson).

Dose-response curves
CM cells were seeded in flat bottom 96-well plates at a density of 2500 cells/well. After 24h, scalar doses of PLX-4032, or an equal volume of DMSO, used as negative control, were added into triplicate wells. Cell viability was evaluated 72h after the addition of PLX-4032 by a standard MTT assay (Life Technologies). Generation of sigmoidal dose-response curves using a four-parameter

nonlinear regression model, and calculation of PLX-4032 IC_{50} values were achieved by the Prism 6.0 software (GraphPad Software).

Immunohistochemical expression of xenograft tumors

Mice bearing Mel 767 parental and/or Mel 767 VR tumors were euthanized at endpoint via CO_2 asphyxiation. Tumors were harvested and fixed in 4% paraformaldehyde overnight. Fixed tissues were dehydrated by submerging in increasing concentrations of ethanol (70-100%), followed by clearing with xylene, and finally infiltrated using Tissue-Tek VIP 6 automated tissue processor prior to embedding. Paraffinized tissues were then embedded in paraffin wax for microtome sectioning. Tissue blocks were sectioned at 4 µm thickness and mounted onto Superfrost® Plus glass microscope slides (Menzel Gläser). Sectioned tumour samples were de-paraffinized followed by enzymatic antigen retrieval and endogenous peroxidase activity blocked by hydrogen peroxide. Sections were then incubated with primary antibodies, mouse-monoclonal anti-human EGFR (31G7, Abcam, 1:100) 36°C for 1 hour, followed by incubation with the appropriate horse radish peroxidase (HRP)-conjugated secondary antibodies at room temperature for 1 hour. Staining was then visualised with 3,3'-diaminutesobenzidine (DAB) and haematoxylin counterstain via the Ventana anti-HQ HRP automated detection system. Slides were imaged using VS120 slide scanners (Olympus) at 40x magnification.

Analysis of TCGA Skin Cutaneous Melanoma (SKCM) cohort

Analysis of TCGA SKCM cohort (dataset TCGA Firehoose Legacy) was performed using https://www.cbioportal.org/ and https://portal.gdc.cancer.gov/ web portals. Only samples with BRAF V600 mutations were considered for the further analysis. Considering the ratio of the expression values of MITF/AXL genes (7), the sampled from the TCGA-SKCM cohort were classified as resistant (R, 32 Samples) or sensitive (S, 93 Samples) . The cut-off of low MITF/AXL ratio was set considering the first quartile of data distribution. The expression values of transcripts for RTK, RTK ligands, and transcription factors were obtained from normalized values downloaded from the web portals. Statistical differences between groups were evaluated by Student’s t test, p-values ≤ 0.05 were considered significant.

Pathway analysis

EGFR-related pathways analysis was conducted with Ingenuity Pathway Analysis software (winter release, December 2023) on genes differentially expressed between the 2 conditions of interest, namely, genes differentially expressed between R vs S in the TCGA SKCM, or between VR and P cells.

Statistical analysis

For RNA-Seq analysis DESeq2 was used for differential expression analysis. Wald-test was employed for hypothesis testing when comparing two groups. A BH adjusted pvalue=0.05 was set. Analysis for significance was performed by parametric Student’s t-test. At least three independent experiments were taken in consideration for each experiment. Values were expressed as mean ± SD. The p-values <0.05 were considered significant.

References

1. Dvorak K, Aggeler B, Palting J, McKelvie P, Ruszkiewicz A, Waring P. Immunohistochemistry with the Anti-Braf V600e (Ve1) Antibody: Impact of Pre-Analytical Conditions and Concordance with DNA Sequencing in Colorectal and Papillary Thyroid Carcinoma. *Pathology* (2014) 46(6):509-17. doi: 10.1097/PAT.0000000000000119.
2. Petti C, Molla A, Vegetti C, Ferrone S, Anichini A, Sensi M. Coexpression of Nrasq61r and Brafv600e in Human Melanoma Cells Activates Senescence and Increases Susceptibility to Cell-

Mediated Cytotoxicity. *Cancer Res* (2006) 66(13):6503-11. Epub 2006/07/05. doi: 10.1158/0008-5472.Can-05-4671.

3. Dugo M, Nicolini G, Tragni G, Bersani I, Tomassetti A, Colonna V, et al. A Melanoma Subtype with Intrinsic Resistance to Braf Inhibition Identified by Receptor Tyrosine Kinases Gene-Driven Classification. *Oncotarget* (2015) 6(7):5118-33.

4. Milione M, Maisonneuve P, Pellegrinelli A, Pusceddu S, Centonze G, Dominoni F, et al. Loss of Succinate Dehydrogenase Subunit B (Sdhb) as a Prognostic Factor in Advanced Ileal Well-Differentiated Neuroendocrine Tumors. *Endocrine* (2017) 57(3):512-7. Epub 2016/12/03. doi: 10.1007/s12020-016-1180-6.

5. Mastorci K, Muraro E, Pasini E, Furlan C, Sigalotti L, Cinco M, et al. Toll-Like Receptor 1/2 and 5 Ligands Enhance the Expression of Cyclin D1 and D3 and Induce Proliferation in Mantle Cell Lymphoma. *PLoS One* (2016) 11(4):e0153823-e. doi: 10.1371/journal.pone.0153823.

6. Ran FA, Hsu PD, Wright J, Agarwala V, Scott DA, Zhang F. Genome Engineering Using the Crispr-Cas9 System. *Nat Protoc* (2013) 8(11):2281-308. Epub 2013/10/24. doi: 10.1038/nprot.2013.143.

7. Müller J, Krijgsman O, Tsoi J, Robert L, Hugo W, Song C, et al. Low Mitf/Axl Ratio Predicts Early Resistance to Multiple Targeted Drugs in Melanoma. *Nature communications* (2014) 5:5712. Epub 2014/12/17. doi: 10.1038/ncomms6712.
