# Supplementary material for: Antibody dependent cellular cytotoxicity-inducing anti-EGFR antibodies as effective therapeutic option for cutaneous melanoma resistant to BRAF inhibitors
Source: Front Immunol. 2024 Mar 6;15:1336566. doi: 10.3389/fimmu.2024.1336566 (PMC10950948; doi:10.3389/fimmu.2024.1336566)

Supplementary Figure 1

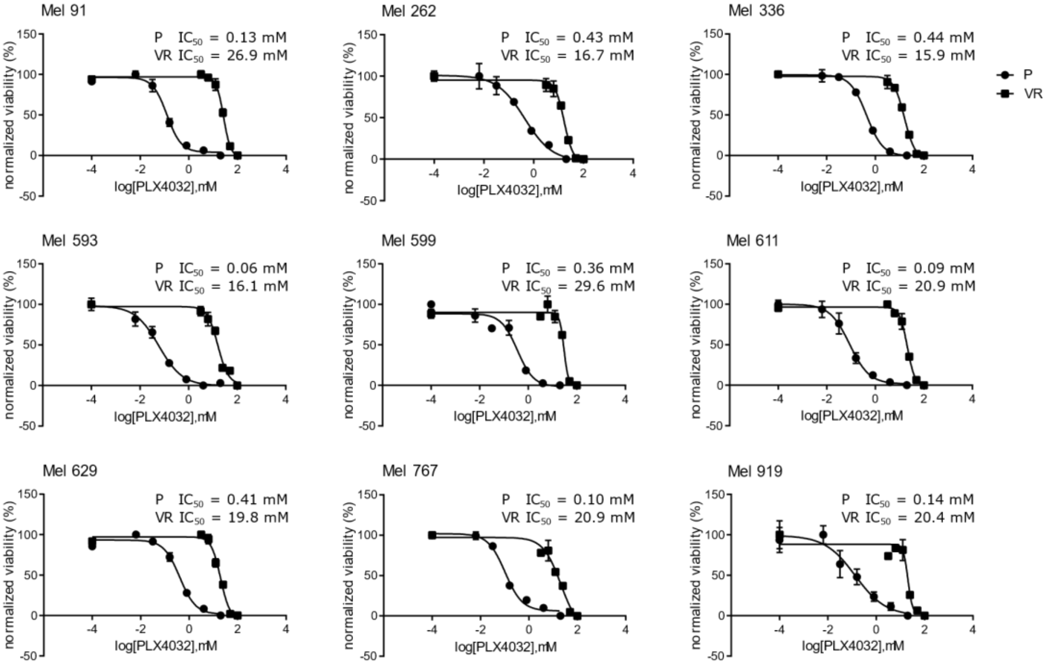

Supplementary Figure 2

A

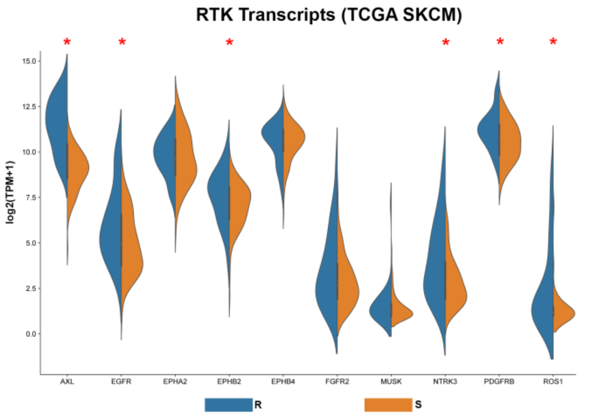

B

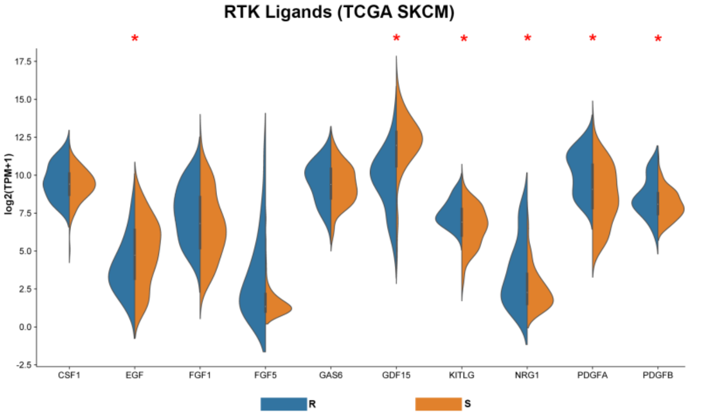

C

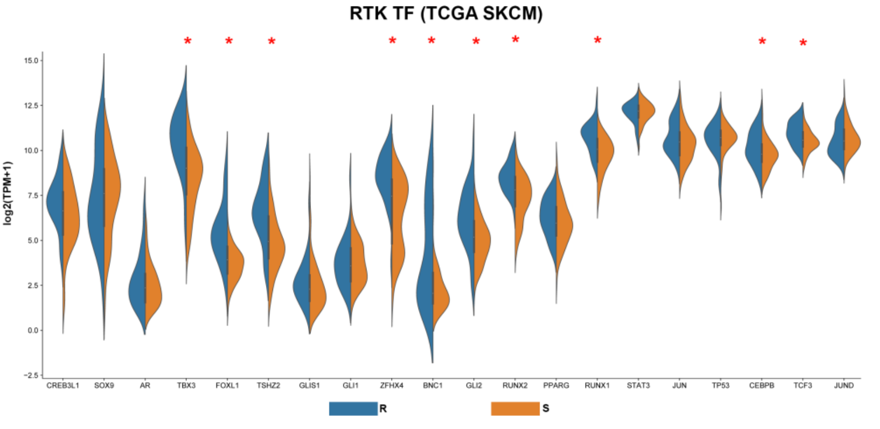

D

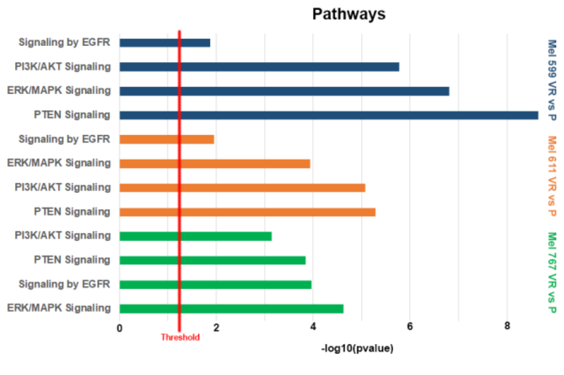

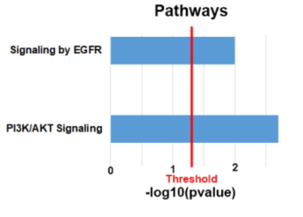

Supplementary Figure 3

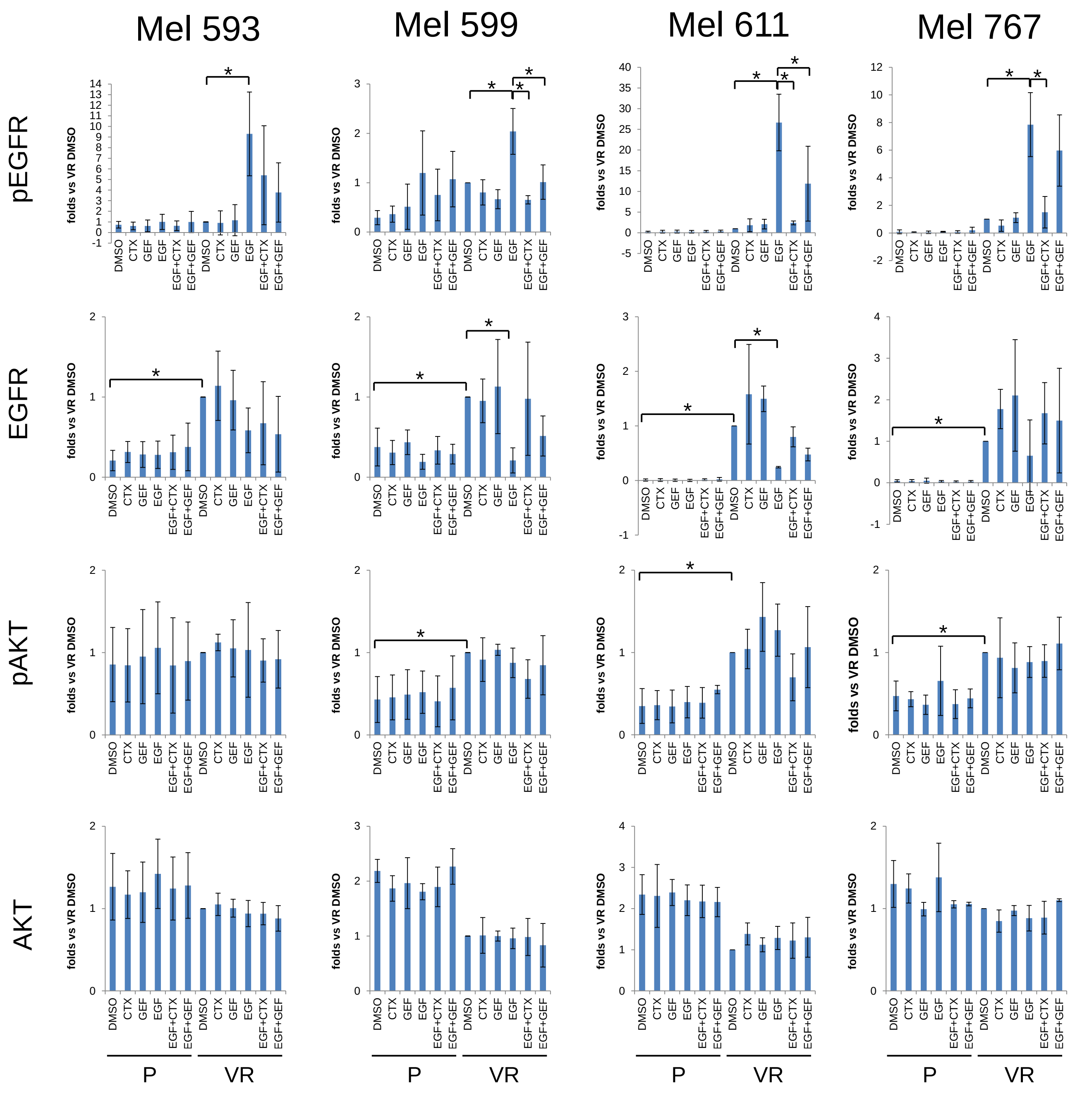

Supplementary Figure 4

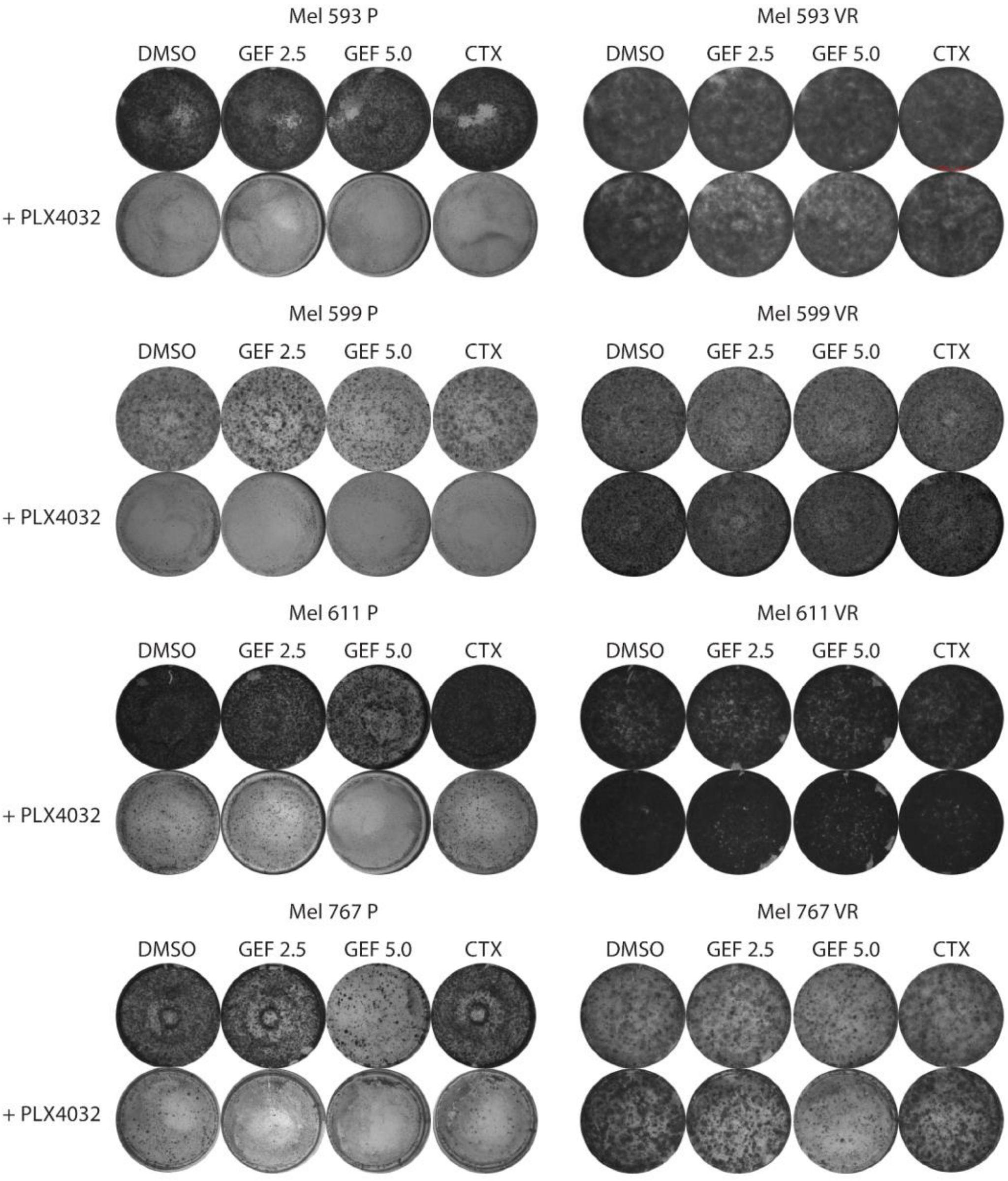

Supplementary Figure 5

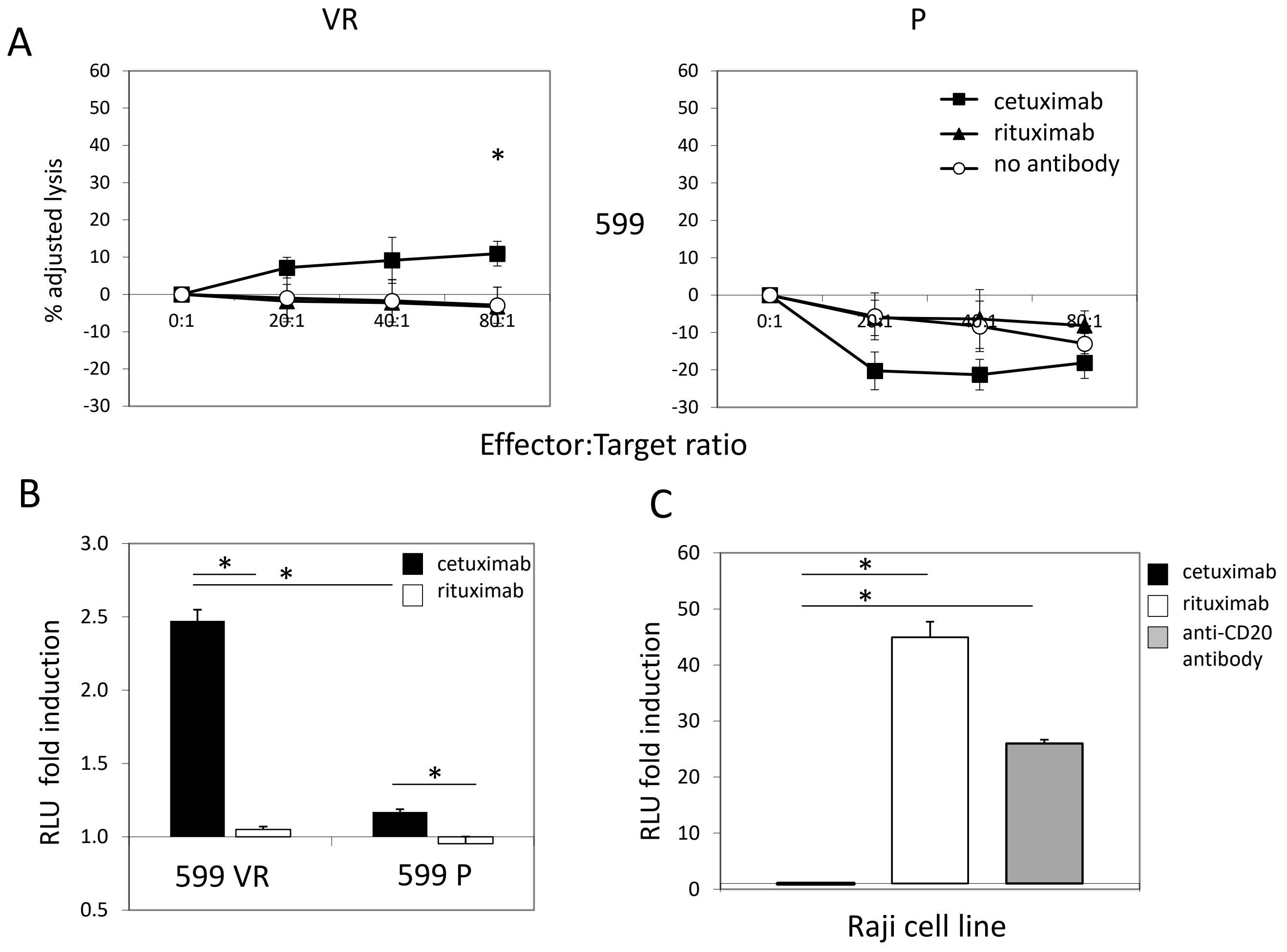

Supplementary Figure 6

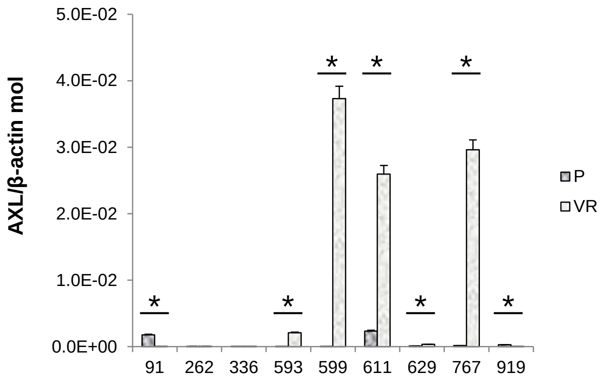

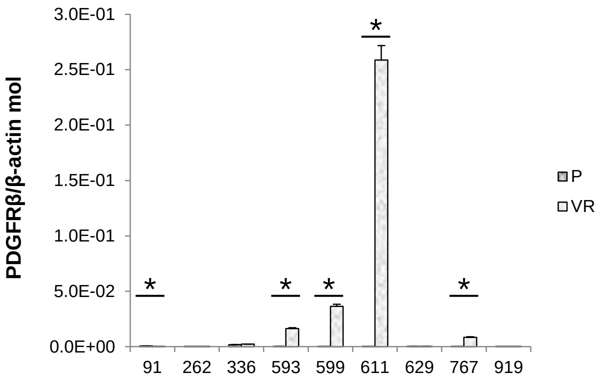

Supplementary Figure 7

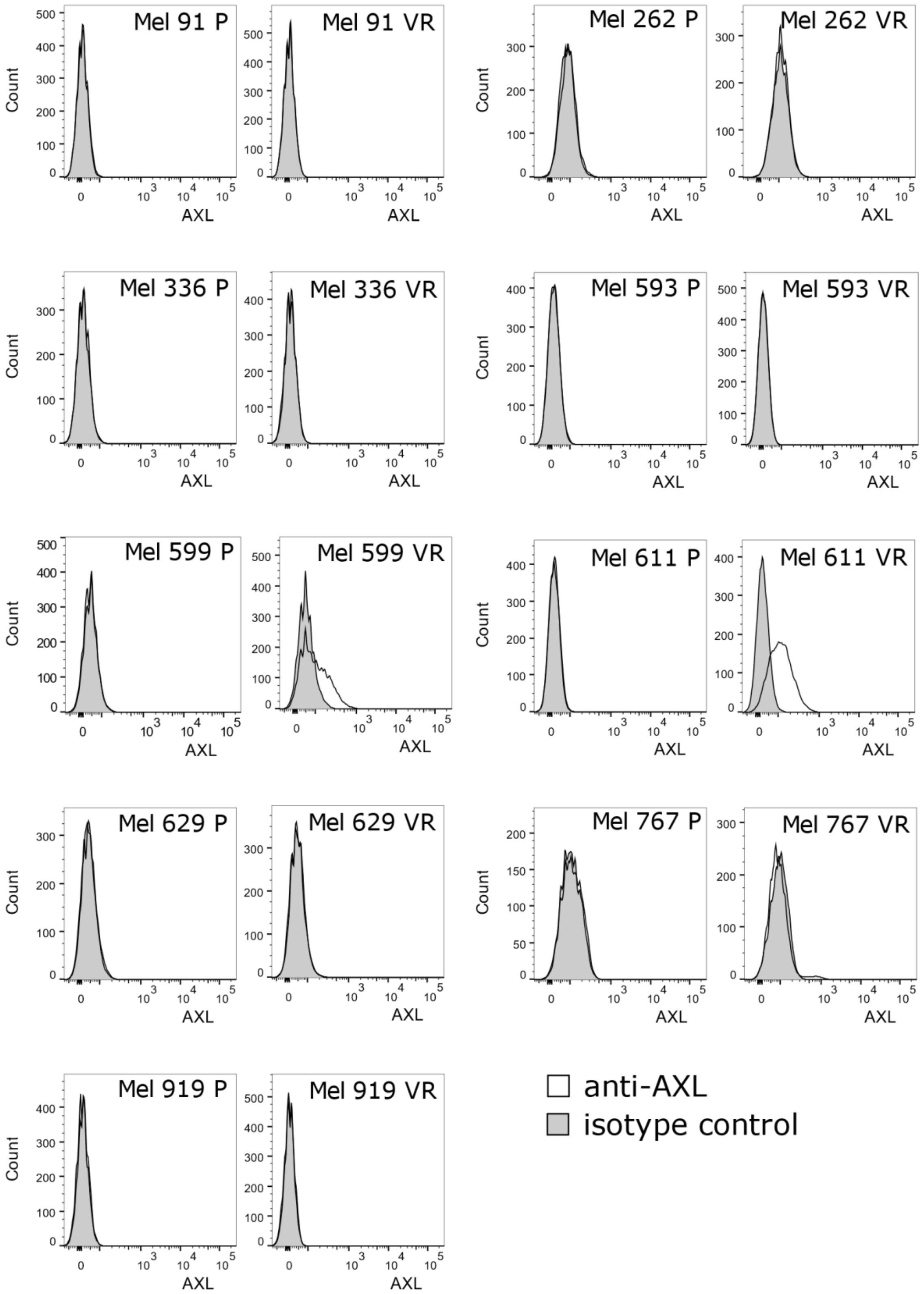

Supplementary Figure 8

A

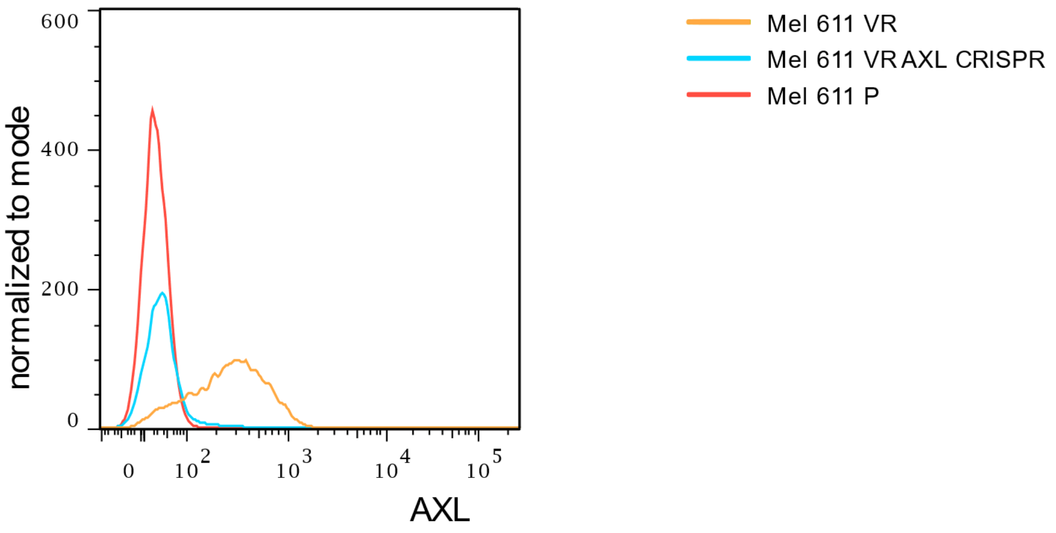

B

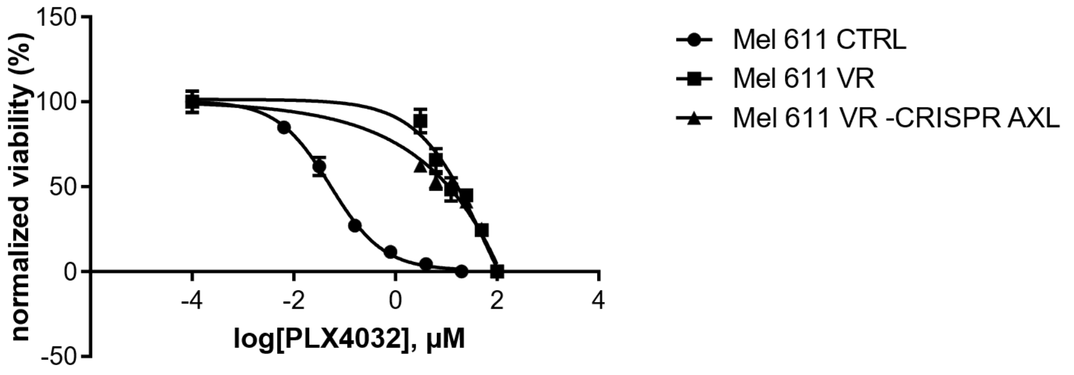

Supplementary Figure 9

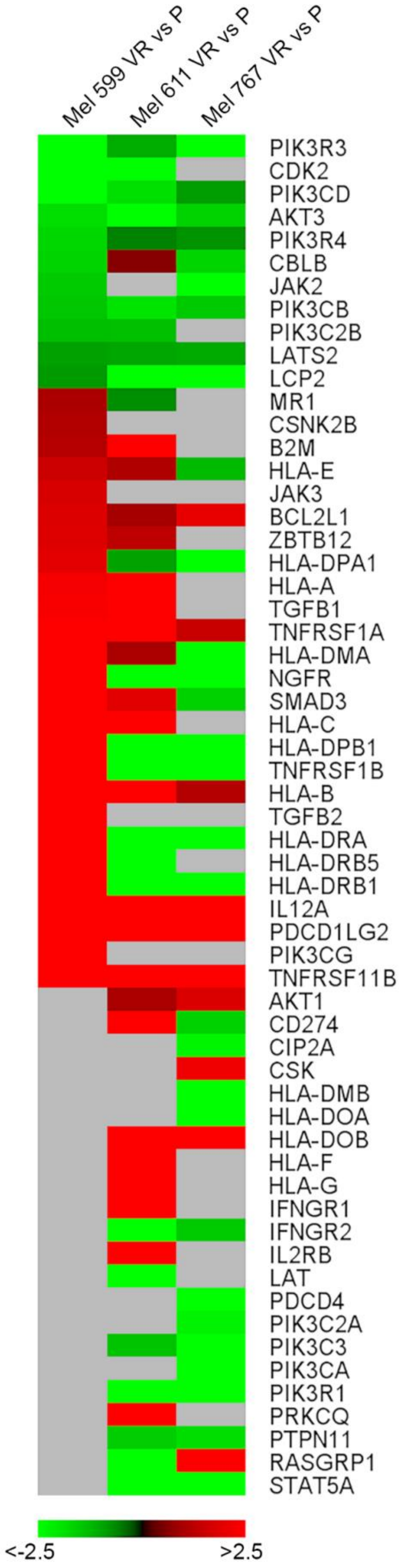

Supplement: Supplementary file 3 [file Image_1.pdf]
