# Supplementary material for: Antibody dependent cellular cytotoxicity-inducing anti-EGFR antibodies as effective therapeutic option for cutaneous melanoma resistant to BRAF inhibitors
Source: Front Immunol. 2024 Mar 6;15:1336566. doi: 10.3389/fimmu.2024.1336566 (PMC10950948; doi:10.3389/fimmu.2024.1336566)
Supplement: Supplementary file 4 [file Table_1.docx]

Supplementary Table 1. Sequencing of BRAF V600 codon of paired P and VR isogenic melanoma cells

| Mel | BRAF status | | Braf mutation | |
| --- | --- | --- | --- | --- |
|  | P | VR | P | VR |
| 91 | wt/mut^1^ | wt/mut | V600E (GAG)^2^ | V600E (GAG) |
| 262 | wt/mut | mut/mut | V600E (GAG) | V600E (GAG) |
| 336 | wt/mut | mut/mut | V600K (AAG) | V600K (AAG) |
| 593 | wt/mut | wt/mut | V600E (GAG) | V600E (GAG) |
| 599 | wt/mut | wt/mut | V600E (GAG) | V600E (GAG) |
| 611 | wt/mut | wt/mut | V600E (GAG) | V600E (GAG) |
| 629 | mut/mut | mut/mut | V600K (AAG) | V600K (AAG) |
| 767 | wt/mut | wt/mut | V600E (GAG) | V600E (GAG) |
| 919 | wt/mut | wt/mut | V600E (GAG) | V600E (GAG) |

^1^ wt = wild type sequence; mut = mutated sequence

^2^ sequence of the mutated codon and respective amino acid substitution
