# Supplementary material for: Antibody dependent cellular cytotoxicity-inducing anti-EGFR antibodies as effective therapeutic option for cutaneous melanoma resistant to BRAF inhibitors
Source: Front Immunol. 2024 Mar 6;15:1336566. doi: 10.3389/fimmu.2024.1336566 (PMC10950948; doi:10.3389/fimmu.2024.1336566)
Supplement: Supplementary file 9 [file Table_6.docx]

Supplementary Table 6. Primer sequences

| **Gene** | **Forward** | **Reverse** | **Reference** |
| --- | --- | --- | --- |
| AR | AAGACGCTTCTACCAGCTCACCAA | TCCCAGAAAGGATCTTGGGCACTT | ([1](#_ENREF_1)) |
| AXL | GTTTGGAGCTGTGATGGAAGGC | CGCTTCACTCAGGAAATCCTCC | ([2](#_ENREF_2)) |
| EGFR | TTCCTCCCAGTGCCTGAA | GGGTTCAGAGGCTGATTGTG | ([3](#_ENREF_3)) |
| EGF | CTTGTCATGCTGCTCCTCCTG | TGCGACTCCTCACATCTCTGC | ([4](#_ENREF_4)) |
| NRG1 | CTACATCTACATCCACCACTGG | TTGCACAAGTATCTCGAGGGGT | ([5](#_ENREF_5)) |
| PDGFRβ | GCTCACACTGACCAACCTCA | GGTGGGATCTGGCACAAAGA | ([6](#_ENREF_6)) |
| β-actin | CGAGCGCGGCTACAGCTT | CCTTAATGTCACGCACGATT | ([7](#_ENREF_7)) |
| BRAF | TGCTTGCTCTGATAGGAAAATG | AGCATCTCAGGGCCAAAAAT | ([8](#_ENREF_8)) |
| NRAS | GGTGAAACCTGTTTGTTGGA | ATGACTTGCTATTATTGATGG | ([9](#_ENREF_9)) |

**References**

1. Qiao L, Tasian GE, Zhang H, Cao M, Ferretti M, Cunha GR, et al. Androgen Receptor Is Overexpressed in Boys with Severe Hypospadias, and Zeb1 Regulates Androgen Receptor Expression in Human Foreskin Cells. *Pediatr Res* (2012) 71(4 Pt 1):393-8. Epub 2012/03/07. doi: 10.1038/pr.2011.49.

2. Gioia R, Leroy C, Drullion C, Lagarde V, Etienne G, Dulucq S, et al. Quantitative Phosphoproteomics Revealed Interplay between Syk and Lyn in the Resistance to Nilotinib in Chronic Myeloid Leukemia Cells. *Blood* (2011) 118(8):2211-21. doi: 10.1182/blood-2010-10-313692.

3. De Cola A, Volpe S, Budani MC, Ferracin M, Lattanzio R, Turdo A, et al. Mir-205-5p-Mediated Downregulation of Erbb/Her Receptors in Breast Cancer Stem Cells Results in Targeted Therapy Resistance. *Cell Death Dis* (2015) 6(7):e1823. Epub 2015/07/17. doi: 10.1038/cddis.2015.192.

4. Zaravinos A, Soufla G, Bizakis J, Spandidos DA. Expression Analysis of Vegfa, Fgf2, Tgfbeta1, Egf and Igf1 in Human Nasal Polyposis. *Oncol Rep* (2008) 19(2):385-91. Epub 2008/01/19.

5. Chua YL, Ito Y, Pole JC, Newman S, Chin SF, Stein RC, et al. The Nrg1 Gene Is Frequently Silenced by Methylation in Breast Cancers and Is a Strong Candidate for the 8p Tumour Suppressor Gene. *Oncogene* (2009) 28(46):4041-52. Epub 2009/10/06. doi: 10.1038/onc.2009.259.

6. Esposito CL, Nuzzo S, Kumar SA, Rienzo A, Lawrence CL, Pallini R, et al. A Combined Microrna-Based Targeted Therapeutic Approach to Eradicate Glioblastoma Stem-Like Cells. *J Control Release* (2016) 238:43-57. doi: https://doi.org/10.1016/j.jconrel.2016.07.032.

7. Fratta E, Sigalotti L, Colizzi F, Covre A, Nicolay HJ, Danielli R, et al. Epigenetically Regulated Clonal Heritability of Cta Expression Profiles in Human Melanoma. *J Cell Physiol* (2010) 223(2):352-8.

8. Dvorak K, Aggeler B, Palting J, McKelvie P, Ruszkiewicz A, Waring P. Immunohistochemistry with the Anti-Braf V600e (Ve1) Antibody: Impact of Pre-Analytical Conditions and Concordance with DNA Sequencing in Colorectal and Papillary Thyroid Carcinoma. *Pathology* (2014) 46(6):509-17. Epub 2014/07/12. doi: 10.1097/PAT.0000000000000119.

9. Petti C, Molla A, Vegetti C, Ferrone S, Anichini A, Sensi M. Coexpression of Nrasq61r and Brafv600e in Human Melanoma Cells Activates Senescence and Increases Susceptibility to Cell-Mediated Cytotoxicity. *Cancer Res* (2006) 66(13):6503-11. Epub 2006/07/05. doi: 10.1158/0008-5472.CAN-05-4671.
